# Supplementary material for: Evaluating the adaptive potential of the European eel: is the immunogenetic status recovering?
Source: PeerJ. 2016 Apr 11;4:e1868. doi: 10.7717/peerj.1868 (PMC4830236; doi:10.7717/peerj.1868)
Supplement: Data S1 [file peerj-04-1868-s001.docx]

**Haplotype sequences and respective segregation sites**

Hap_1 TGACCTATCAACATTTCCATACAAACGAAGCTATATCTCCATAACGCCAGCCGAAAGCATGCTATAATATCGAGCTA

Hap_2 ...........T..................................................C..............

Hap_3 ............................................................................G

Hap_4 ...........T.................................................................

Hap_5 ...........T................G................................................

Hap_6 ...........T..............................G..................................

Hap_7 ...........T.................A................................C..............

Hap_8 ...........T...........G.....A................................C..............

Hap_9 ...........T..........G..................................T....C..............

Hap_10 ..................................T.................................G........

Hap_11 ..G........T..................................................C..............

Hap_12 ...........T............................................AT...................

Hap_13 ...............C............................................................G

Hap_14 ..........................................................G..................

Hap_15 ...........T..................................................C...G..........

Hap_16 ..........................A.................................................G

Hap_17 ...........T..................................................C.C............

Hap_18 ...........T..C..............................................................

Hap_19 .......................................................G.....................

Hap_20 ...........................G.................................................

Hap_21 ............................G................................................

Hap_22 ....................................................A........................

Hap_23 ...........T.....................................A...........................

Hap_24 ...........T..............................................................T..

Hap_25 ..........G..................................................................

Hap_26 ...........T........................................A.........C..............

Hap_27 ......................................T......................................

Hap_28 ...........TC................................................................

Hap_29 ..............................................T..............................

Hap_30 ...................................................................C.........

Hap_31 ...............................................T.............................

Hap_32 ...........T........G.........................................C..............

Hap_33 ...........T.......C..........................................C..............

Hap_34 ...........T..................T...............................C..............

Hap_35 ...........T.............................................T....C..............

Hap_36 ...........T................G.................................C..............

Hap_37 ...........T..................................................C...........T..

Hap_38 ...........T..................G..............................................

Hap_39 ...........T....A............................................................

Hap_40 ...........T........................................A........................

Hap_41 .A...........................................................................

Hap_42 ......................................................G......................

Hap_43 ................................T............................................

Hap_44 .............................................................T...............

Hap_45 .................................................................G...........

Hap_46 ....................................T........................................

Hap_47 .........................................................T..................G

Hap_48 ..G.........................................................................G

Hap_49 ...........................................G................................G

Hap_50 .....................T......................................................G

Hap_51 .......................................T..................G..................

Hap_52 ....T.....................................................G..................

Hap_53 ...............................C...................................C.........

Hap_54 ...........T..................................T......G.......................

Hap_55 ..........................................G...........................A......

Hap_56 ...................................................T.........................

Hap_57 .....................................................................C.......

Hap_58 ..........G...............................................G..................

Hap_59 ........G..T....................................C....G........C..............

Hap_60 ...........................................................................A.

Hap_61 ...........T............G.....................................C..............

Hap_62 .....................................................G.............C.........

Hap_63 ...........T.............................CG..................................

Hap_64 ...........T.......................C................A.........C..............

Hap_65 ....................................................................G........

Hap_66 ..G........T.................................................................

Hap_67 .................T........................................G..................

Hap_68 ...........T.........................C.......................................

Hap_69 ...........T.....................C............................C..............

Hap_70 ...........T................................................A................

Hap_71 ...........T.................A................................C..........A...

Hap_72 ..........................A..................................................

Hap_73 ...........T.....................................A......................G....

Hap_74 ...........T...........G.......................T..............C..............

Hap_75 ..................G.....................................A...................G

Hap_76 ...........T..................................................C........A.....

Hap_77 ...........T................................T.................C..............

Hap_78 ...........T................................................A.C..............

Hap_79 ...........T..................................................C.......T......

Hap_80 ....T......T..............................................G..................

Hap_81 ..................G.........................................................G

Hap_82 ....T.....................A...............................G..................

Hap_83 ..................................................T..........................

Hap_84 .........T...................................................................

Hap_85 ...T.......T..................................................C..............

Hap_86 C............................................................................

Hap_87 ...........................................................C.................

Hap_88 .....C......................................................A..G............G

Hap_89 .............G..........C....................................................

Hap_90 ......G....T..................................................C..............

Hap_91 ......................................................................A.....G

Hap_92 ..........................A...............................G..................

Hap_93 ........................................G....................................

Hap_94 ...................................................................CG........

Hap_95 ...........T.............................................T...................

Hap_96 ...........T..............A...................................C..............

Hap_97 ......................................................................T......

Hap_98 .................A...........................................................

Hap_99 .........................T................................G..................

Hap_100 ...........T................G....................A...........................

Hap_101 ...........T.................................A...............................

Hap_102 .......C...T..................................................C..............

**Haplotypes distribution amongst sampled locations**

[Hap_1: 215 AD2010_01 AD2010_05 AD2010_07 AD2010_11 AD2010_13 AD2010_14 AD2010_16 AD2010_17 AD2010_18 AD2010_19 AD2010_20 AD2010_25 AD2010_26 AD2010_29 AD2010_30 AD2010_31 AD2010_38 AD2010_41 AD2010_42 AD2010_46 AD2010_48 AD2010_50 AD2010_51 AD2010_53 AD2010_58 AD2010_60 AD2010_61 AD2010_63 AD2010_64 AD2010_68 AD2010_69 AD2010_70 AD2010_71 AD2010_77 AD2010_80 AD2010_81 AD2010_103 AD2010_105 AD2010_109 AD2010_111 AD2010_116 AD2010_131 AD2010_132 AD2010_134 AD2010_137 AD2010_138 AD2010_141 AD2010_142 AD2010_147 AD2010_159 AD2010_86 AD2010_88 AD2010_93 AD2010_98 AD2010_89 AD2011_78 AD2011_79 AD2011_109 AD2011_136 AD2011_138 AD2011_142 AD2011_157 AD2011_159 AD2011_17 AD2011_24 AD2011_30 AD2011_32 AD2011_34 AD2011_3 AD2011_41 AD2011_42 AD2011_43 AD2011_48 AD2011_52 AD2011_53 AD2011_54 AD2011_56 AD2011_61 AD2011_62 AD2011_65 AD2011_66 AD2011_69 AD2011_6 AD2011_82 AD2011_85 AD2011_87 AD2011_89 AD2011_90 AD2011_97 AD2011_9 AD2012_06 AD2012_08 AD2012_102 AD2012_103 AD2012_11 AD2012_118 AD2012_119 AD2012_120 AD2012_121 AD2012_132 AD2012_134 AD2012_135 AD2012_136 AD2012_138 AD2012_139 AD2012_147 AD2012_15 AD2012_152 AD2012_19 AD2012_23 AD2012_30 AD2012_32 AD2012_34 AD2012_39 AD2012_61 AD2012_72 AD2012_73 AD2012_75 AD2012_76 AD2012_79 AD2012_80 AD2012_83 AD2012_84 AD2012_85 AD2012_86 AD2012_95 AD2012_97 LC09 LC98 LC79 Q09 Q47 Q52 Q48 Q03 Q51 Q39 Q05 Q02 BU19 BU13 BU01 BU54 BU06 BU52 BU38 BT10 BT12 BT20 BT16 LC12 BL06 SLC03 SLC01 SLC11 SLCO9 LL50 LL37 SLB08 SLB16 GL03 GL62 GL01 BL05 BL11 Dk12 Dk02 Dk28 Dk08 FI15 FI16 FI18 FI05 P12 P19 P21 P30 WK171 Wk236 Wk287 Wk289 P11 DK25 BT09 BT11 BU07 BU20 BU35 LC10 SLB02 SLB03 CH-B-IRL1 CH-B-IRL11 CH-B-IRL3 CH-B-IRL8 CH-B-IRL9 CH-B-NIRL4 CH-B-NIRL6 CH-B-NIRL9 CH-C-NIRL2 CH-E-GER1 CH-E-GER2 CH-O-SPA4 CH-T-ENG10 CH-T-ENG2 CH-T-ENG5 CH-V-SWE10 CH-V-SWE11 CH-V-SWE15 CH-V-SWE16 CH-V-SWE6 CH-V-SWE8 CH-V-SWE9 CH-W-ENG1 CH-W-ENG3]

[Hap_2: 143 AD2010_02 AD2010_04 AD2010_15 AD2010_21 AD2010_22 AD2010_24 AD2010_28 AD2010_32 AD2010_39 AD201043 AD2010_44 AD2010_49 AD2010_56 AD201066 AD2010_78 AD2010_82 AD2010_112 AD2010_113 AD2010_117 AD2010_120 AD2010_127 AD2010_136 AD2010_149 AD2010_156 AD2010_162 AD2010_92 AD2010_94 AD2011_101 AD2011_84 AD2011_102 AD2011_107 AD2011_111 AD2011_12 AD2011_132 AD2011_134 AD2011_137 AD2011_140 AD2011_143 AD2011_146 AD2011_14 AD2011_19 AD2011_20 AD2011_27 AD2011_2 AD2011_35 AD2011_40 AD2011_45 AD2011_47 AD2011_4 AD2011_51 AD2011_67 AD2011_70 AD2011_93 AD2011_94 AD2011_99 AD2012_09 AD2012_101 AD2012_107 AD2012_113 AD2012_114 AD2012_115 AD2012_117 AD2012_14 AD2012_143 AD2012_16 AD2012_22 AD2012_29 AD2012_40 AD2012_44 AD2012_59 AD2012_60 AD2012_66 AD2012_68 AD2012_70 AD2012_71 AD2012_77 AD2012_90 AD2012_92 AD2012_93 LC11 LC03 LC77 LC04 Q50 BU22 BU11 BT02 BT05 LC07 LC63 BT07 BL09 BL10 LL25 LL52 LL09 LL17 LL05 SLB14 GL02 GL58 GL53 Dk19 Dk09 FI02 FI22 FI31 FI06 FI08 P17 P08 Wk213 Wk229 Wk237 Wk249 Wk267 Wk275 Wk290 P28 P07 P23 BL01 BT18 BU48 BU55 LC02 LC06 SLB10 SLB13 CH-B-IRL14 CH-B-IRL15 CH-B-IRL4 CH-B-IRL6 CH-B-IRL7 CH-B-NIRL1 CH-B-NIRL10 CH-B-NIRL5 CH-C-NIRL3 CH-E-GER3 CH-T-ITA4 CH-T-ITA5 CH-V-SWE5 CH-W-ENG5]

[Hap_3: 36 AD2010_03 AD2010_10 AD2010_40 AD2010_67 AD2010_73 AD2010_139 AD2010_143 AD2010_90 AD2011_73 AD2011_7 AD2011_141 AD2011_15 AD2011_29 AD2011_36 AD2011_80 AD2012_04 AD2012_133 AD2012_142 AD2012_145 AD2012_149 AD2012_82 BT19 BL13 SLC02 SLB15 SLB07 Dk23 Dk29 FI09 P20 Wk244 P22 P24 SLC10 CH-V-SWE14 CH-V-SWE2]

[Hap_4: 87 AD2010_06 AD2010_08 AD2010_09 AD2010_23 AD2010_27 AD2010_34 AD2010_36 AD2010_37 AD2010_52 AD2010_57 AD2010_75 AD2010_79 AD2010_101 AD2010_102 AD2010_104 AD2010_106 AD2010_107 AD2010_118 AD2010_119 AD2010_121 AD2010_129 AD2010_163 AD2010_85 AD2010_91 AD2011_10 AD2011_110 AD2011_103 AD2011_106 AD2011_153 AD2011_21 AD2011_26 AD2011_33 AD2011_39 AD2011_44 AD2011_50 AD2011_57 AD2011_83 AD2011_91 AD2011_92 AD2011_96 AD2012_05 AD2012_106 AD2012_110 AD2012_111 AD2012_128 AD2012_137 AD2012_144 AD2012_18 AD2012_27 AD2012_33 AD2012_45 AD2012_64 AD2012_74 AD2012_81 AD2012_96 LC86 LC58 LC01 LC05 Q11 Q46 BT06 BL07 BL04 LL42 LL04 LL11 SLB05 SLB11 SLB06 GL64 BL02 FI11 FI23 P25 Wk210 Wk238 Wk245 SLB12 CH-E-GER5 CH-O-SPA2 CH-O-SPA5 CH-T-ENG1 CH-T-ITA1 CH-V-SWE1 CH-VILL1 CH-VILL4]

[Hap_5: 6 AD2010_12 AD2010_153 AD2012_104 AD2012_69 CH-B-NIRL7 CH-W-ENG2]

[Hap_6: 10 AD2010_33 AD2010_154 AD2011_38 AD2012_108 AD2012_28 BT13 BL08 GL52 FI17 CH-O-SPA3]

[Hap_7: 11 AD2010_35 AD2010_110 AD2010_99 AD2011_118 AD2011_139 AD2011_18 AD2011_60 AD2012_41 BT15 CH-B-IRL10 CH-C-NIRL1]

[Hap_8: 1 AD2010_45]

[Hap_9: 1 AD2010_47]

[Hap_10: 1 AD2010_54]

[Hap_11: 7 AD2010_55 AD2011_130 AD2011_13 AD2012_78 LL16 Dk06 FI32]

[Hap_12: 3 AD2010_62 AD2010_144 AD2012_57]

[Hap_13: 1 AD2010_65]

[Hap_14: 35 AD2010_100 AD2010_114 AD2010_135 AD2010_151 AD2010_157 AD2010_164 AD2010_87 AD2010_96 AD2011_104 AD2011_81 AD2011_105 AD2011_108 AD2011_37 AD2011_49 AD2012_122 AD2012_140 AD2012_141 AD2012_146 AD2012_21 AD2012_38 Q07 Q17 LL43 Dk21 Dk26 FI20 FI21 FI25 CH-B-IRL13 CH-E-GER4 CH-T-ENG6 CH-T-ENG8 CH-V-SWE13 CH-V-SWE7 CH-VILL3]

[Hap_15: 1 AD2010_115]

[Hap_16: 1 AD2010_122]

[Hap_17: 1 AD2010_124]

[Hap_18: 1 AD2010_125]

[Hap_19: 1 AD2010_126]

[Hap_20: 1 AD2010_128]

[Hap_21: 1 AD2010_130]

[Hap_22: 5 AD2010_133 AD2010_148 BU24 GL57 DK30]

[Hap_23: 9 AD2010_140 AD2011_100 AD2011_98 AD2011_86 AD2012_105 AD2012_131 LL15 GL61 P18]

[Hap_24: 5 AD2010_145 AD2011_158 AD2011_64 FI01 SLC08]

[Hap_25: 1 AD2010_146]

[Hap_26: 2 AD2010_150 AD2010_160]

[Hap_27: 3 AD2010_152 AD2011_11 AD2012_87]

[Hap_28: 1 AD2010_155]

[Hap_29: 2 AD2010_161 Dk07]

[Hap_30: 3 AD2010_84 AD2012_42 LC83]

[Hap_31: 1 AD2010_97]

[Hap_32: 1 AD2011_129]

[Hap_33: 1 AD2011_46]

[Hap_34: 1 AD2011_63]

[Hap_35: 2 AD2011_76 CH-V-SWE4]

[Hap_36: 1 AD2011_8]

[Hap_37: 1 AD2011_133]

[Hap_38: 1 AD2011_112]

[Hap_39: 1 AD2011_155]

[Hap_40: 1 AD2011_75]

[Hap_41: 1 AD2011_131]

[Hap_42: 1 AD2011_135]

[Hap_43: 1 AD2011_16]

[Hap_44: 4 AD2011_71 AD2012_148 Dk03 CH-T-ITA3]

[Hap_45: 1 AD2011_74]

[Hap_46: 2 AD2011_22 AD2011_28]

[Hap_47: 1 AD2011_1]

[Hap_48: 1 AD2011_59]

[Hap_49: 1 AD2011_55]

[Hap_50: 1 AD2011_77]

[Hap_51: 1 AD2011_58]

[Hap_52: 3 AD2011_68 AD2012_150 BT17]

[Hap_53: 1 AD2011_5]

[Hap_54: 1 AD2011_23]

[Hap_55: 2 AD2012_02 GL55]

[Hap_56: 2 AD2012_03 CH-B-NIRL12]

[Hap_57: 1 AD2012_112]

[Hap_58: 1 AD2012_116]

[Hap_59: 1 AD2012_12]

[Hap_60: 1 AD2012_125]

[Hap_61: 2 AD2012_126 AD2012_20]

[Hap_62: 1 AD2012_17]

[Hap_63: 1 AD2012_24]

[Hap_64: 1 AD2012_25]

[Hap_65: 1 AD2012_31]

[Hap_66: 1 AD2012_48]

[Hap_67: 1 AD2012_62]

[Hap_68: 1 AD2012_63]

[Hap_69: 1 AD2012_65]

[Hap_70: 2 AD2012_67 CH-V-SWE3]

[Hap_71: 1 AD2012_88]

[Hap_72: 1 AD2012_89]

[Hap_73: 2 AD2012_94 BT04]

[Hap_74: 1 Q01]

[Hap_75: 1 BU12]

[Hap_76: 1 BT08]

[Hap_77: 3 SLC05 DK11 SLB01]

[Hap_78: 1 SLC04]

[Hap_79: 2 SLB09 CH-B-NIRL2]

[Hap_80: 1 GL63]

[Hap_81: 2 GL51 Dk17]

[Hap_82: 1 GL60]

[Hap_83: 1 Dk24]

[Hap_84: 1 Dk05]

[Hap_85: 1 FI14]

[Hap_86: 1 WK203]

[Hap_87: 1 Wk252]

[Hap_88: 1 Dk04]

[Hap_89: 1 P27]

[Hap_90: 1 P16]

[Hap_91: 1 LC76]

[Hap_92: 1 CH-B-IRL12]

[Hap_93: 1 CH-B-IRL5]

[Hap_94: 1 CH-B-NIRL11]

[Hap_95: 1 CH-B-NIRL3]

[Hap_96: 1 CH-B-NIRL8]

[Hap_97: 1 CH-T-ENG3]

[Hap_98: 1 CH-T-ENG4]

[Hap_99: 1 CH-T-ENG7]

[Hap_100: 1 CH-T-ENG9]

[Hap_101: 1 CH-V-SWE12]

[Hap_102: 1 CH-W-ENG4]
