# Supplementary material for: Evaluating the adaptive potential of the European eel: is the immunogenetic status recovering?
Source: PeerJ. 2016 Apr 11;4:e1868. doi: 10.7717/peerj.1868 (PMC4830236; doi:10.7717/peerj.1868)
Supplement: Data S2 [file peerj-04-1868-s002.docx]

**Allelic frequencies at each locus**

Locus: 1

Pop Alleles Genes

----------------------------------------------------------------------------------------------------------------- -----

1 2 3 4 5 6 7 8 9 10 11 12 13 14 15 16 17 18 19

Ad2010 0.013 0.007 0.013 0.076 0.189 0.079 0.285 0.116 0.103 0.036 0.036 0.003 0.013 0.013 0.017 0.000 0.000 0.000 0.000 302

Ad2011 0.026 0.000 0.004 0.096 0.213 0.091 0.243 0.061 0.100 0.083 0.030 0.013 0.017 0.004 0.004 0.000 0.009 0.004 0.000 230

ad2012 0.000 0.004 0.013 0.079 0.200 0.096 0.254 0.125 0.087 0.071 0.013 0.013 0.008 0.013 0.000 0.021 0.000 0.004 0.000 240

LC 0.000 0.000 0.025 0.075 0.250 0.050 0.225 0.050 0.150 0.075 0.050 0.000 0.000 0.000 0.025 0.025 0.000 0.000 0.000 40

BT 0.059 0.000 0.000 0.029 0.265 0.088 0.265 0.088 0.118 0.088 0.000 0.000 0.000 0.000 0.000 0.000 0.000 0.000 0.000 34

Q 0.000 0.000 0.038 0.000 0.192 0.000 0.538 0.038 0.077 0.077 0.038 0.000 0.000 0.000 0.000 0.000 0.000 0.000 0.000 26

CHWENG 0.000 0.000 0.000 0.000 0.100 0.100 0.300 0.100 0.000 0.200 0.100 0.100 0.000 0.000 0.000 0.000 0.000 0.000 0.000 10

CHVILL 0.000 0.000 0.000 0.000 0.167 0.333 0.333 0.000 0.000 0.000 0.167 0.000 0.000 0.000 0.000 0.000 0.000 0.000 0.000 6

CHSWE 0.000 0.000 0.000 0.031 0.312 0.094 0.219 0.094 0.000 0.125 0.062 0.062 0.000 0.000 0.000 0.000 0.000 0.000 0.000 32

CHITA 0.000 0.000 0.000 0.000 0.250 0.000 0.375 0.000 0.000 0.375 0.000 0.000 0.000 0.000 0.000 0.000 0.000 0.000 0.000 8

CHBIRL 0.107 0.000 0.036 0.107 0.179 0.000 0.393 0.036 0.036 0.071 0.000 0.000 0.000 0.036 0.000 0.000 0.000 0.000 0.000 28

BNIRL 0.000 0.000 0.042 0.042 0.083 0.083 0.375 0.125 0.000 0.208 0.042 0.000 0.000 0.000 0.000 0.000 0.000 0.000 0.000 24

SPA 0.000 0.000 0.000 0.125 0.375 0.000 0.250 0.125 0.000 0.000 0.000 0.000 0.000 0.125 0.000 0.000 0.000 0.000 0.000 8

CNIRL 0.100 0.000 0.000 0.300 0.200 0.000 0.200 0.000 0.000 0.000 0.100 0.100 0.000 0.000 0.000 0.000 0.000 0.000 0.000 10

GER 0.000 0.100 0.000 0.000 0.600 0.100 0.100 0.000 0.000 0.000 0.100 0.000 0.000 0.000 0.000 0.000 0.000 0.000 0.000 10

BU 0.000 0.000 0.000 0.000 0.250 0.094 0.188 0.031 0.188 0.156 0.062 0.000 0.031 0.000 0.000 0.000 0.000 0.000 0.000 32

BL 0.000 0.000 0.000 0.045 0.182 0.045 0.136 0.045 0.409 0.045 0.000 0.000 0.000 0.000 0.045 0.000 0.045 0.000 0.000 22

SLC 0.000 0.000 0.000 0.071 0.143 0.071 0.286 0.071 0.143 0.000 0.143 0.000 0.000 0.071 0.000 0.000 0.000 0.000 0.000 14

LL 0.000 0.000 0.077 0.038 0.154 0.077 0.192 0.077 0.154 0.038 0.038 0.077 0.000 0.000 0.077 0.000 0.000 0.000 0.000 26

SLB 0.000 0.000 0.033 0.033 0.367 0.200 0.133 0.100 0.133 0.000 0.000 0.000 0.000 0.000 0.000 0.000 0.000 0.000 0.000 30

GL 0.000 0.000 0.000 0.083 0.167 0.042 0.292 0.208 0.125 0.000 0.042 0.000 0.042 0.000 0.000 0.000 0.000 0.000 0.000 24

DK 0.000 0.000 0.000 0.053 0.289 0.184 0.184 0.079 0.132 0.026 0.026 0.000 0.000 0.000 0.026 0.000 0.000 0.000 0.000 38

FI 0.025 0.000 0.000 0.075 0.325 0.025 0.300 0.050 0.075 0.075 0.025 0.025 0.000 0.000 0.000 0.000 0.000 0.000 0.000 40

PT 0.059 0.000 0.000 0.059 0.118 0.206 0.147 0.029 0.118 0.118 0.088 0.029 0.000 0.000 0.029 0.000 0.000 0.000 0.000 34

WK 0.000 0.000 0.000 0.088 0.235 0.059 0.324 0.059 0.029 0.088 0.000 0.059 0.000 0.029 0.029 0.000 0.000 0.000 0.000 34

CHTENG 0.050 0.050 0.050 0.050 0.000 0.050 0.200 0.150 0.200 0.100 0.050 0.000 0.000 0.000 0.000 0.000 0.000 0.000 0.050 20

Locus: 2

------------------

Pop Alleles Genes

----------------------------------------------------------------------------------------------------------------------- -----

1 2 3 4 5 6 7 8 9 10 11 12 13 14 15 16 17 18 19 20

Ad2010 0.003 0.000 0.010 0.033 0.040 0.043 0.147 0.203 0.147 0.100 0.107 0.040 0.053 0.023 0.007 0.013 0.010 0.013 0.003 0.003 300

Ad2011 0.000 0.013 0.017 0.030 0.057 0.057 0.178 0.157 0.096 0.122 0.126 0.061 0.022 0.009 0.004 0.022 0.013 0.000 0.009 0.009 230

ad2012 0.004 0.008 0.004 0.033 0.041 0.054 0.149 0.207 0.153 0.120 0.103 0.066 0.037 0.000 0.004 0.000 0.008 0.004 0.004 0.000 242

LC 0.000 0.000 0.025 0.025 0.025 0.025 0.200 0.175 0.150 0.050 0.150 0.025 0.050 0.000 0.050 0.000 0.025 0.000 0.025 0.000 40

BT 0.000 0.000 0.000 0.000 0.088 0.059 0.059 0.176 0.088 0.176 0.147 0.000 0.118 0.029 0.000 0.029 0.000 0.000 0.029 0.000 34

Q 0.000 0.000 0.000 0.000 0.038 0.154 0.154 0.154 0.077 0.077 0.192 0.000 0.077 0.000 0.000 0.000 0.000 0.000 0.077 0.000 26

CHWENG 0.000 0.000 0.000 0.000 0.000 0.200 0.000 0.200 0.100 0.200 0.300 0.000 0.000 0.000 0.000 0.000 0.000 0.000 0.000 0.000 10

CHVILL 0.000 0.000 0.000 0.167 0.000 0.000 0.167 0.000 0.167 0.000 0.333 0.167 0.000 0.000 0.000 0.000 0.000 0.000 0.000 0.000 6

CHSWE 0.000 0.000 0.000 0.062 0.031 0.062 0.094 0.156 0.125 0.125 0.125 0.062 0.094 0.031 0.000 0.000 0.000 0.031 0.000 0.000 32

CHITA 0.000 0.000 0.000 0.000 0.000 0.000 0.000 0.750 0.250 0.000 0.000 0.000 0.000 0.000 0.000 0.000 0.000 0.000 0.000 0.000 8

CHBIRL 0.000 0.000 0.000 0.107 0.000 0.071 0.214 0.179 0.214 0.036 0.143 0.000 0.000 0.000 0.000 0.000 0.000 0.000 0.000 0.036 28

BNIRL 0.000 0.000 0.000 0.042 0.000 0.083 0.083 0.250 0.083 0.125 0.250 0.083 0.000 0.000 0.000 0.000 0.000 0.000 0.000 0.000 24

SPA 0.000 0.000 0.000 0.000 0.000 0.125 0.000 0.250 0.250 0.000 0.125 0.000 0.125 0.125 0.000 0.000 0.000 0.000 0.000 0.000 8

CNIRL 0.000 0.000 0.000 0.000 0.000 0.000 0.200 0.000 0.300 0.000 0.300 0.200 0.000 0.000 0.000 0.000 0.000 0.000 0.000 0.000 10

GER 0.000 0.000 0.000 0.100 0.000 0.000 0.300 0.200 0.100 0.000 0.000 0.200 0.000 0.000 0.000 0.100 0.000 0.000 0.000 0.000 10

BU 0.000 0.000 0.000 0.031 0.000 0.000 0.031 0.281 0.125 0.031 0.250 0.094 0.094 0.031 0.031 0.000 0.000 0.000 0.000 0.000 32

BL 0.000 0.000 0.000 0.000 0.045 0.091 0.227 0.136 0.273 0.136 0.000 0.000 0.000 0.000 0.000 0.000 0.000 0.091 0.000 0.000 22

SLC 0.000 0.000 0.000 0.071 0.071 0.000 0.214 0.143 0.071 0.071 0.286 0.071 0.000 0.000 0.000 0.000 0.000 0.000 0.000 0.000 14

LL 0.000 0.000 0.000 0.042 0.000 0.042 0.167 0.208 0.125 0.292 0.042 0.000 0.000 0.000 0.042 0.000 0.000 0.042 0.000 0.000 24

SLB 0.000 0.000 0.000 0.000 0.133 0.133 0.100 0.167 0.133 0.067 0.100 0.000 0.033 0.000 0.033 0.067 0.033 0.000 0.000 0.000 30

GL 0.000 0.000 0.000 0.000 0.038 0.077 0.192 0.231 0.038 0.077 0.077 0.038 0.154 0.000 0.000 0.077 0.000 0.000 0.000 0.000 26

DK 0.000 0.000 0.000 0.000 0.026 0.079 0.158 0.237 0.158 0.079 0.105 0.053 0.053 0.000 0.000 0.000 0.026 0.026 0.000 0.000 38

FI 0.000 0.025 0.000 0.025 0.075 0.075 0.125 0.075 0.125 0.100 0.150 0.075 0.075 0.025 0.000 0.000 0.000 0.050 0.000 0.000 40

PT 0.000 0.000 0.000 0.000 0.125 0.031 0.219 0.094 0.125 0.156 0.156 0.000 0.031 0.000 0.000 0.031 0.000 0.031 0.000 0.000 32

WK 0.000 0.000 0.000 0.059 0.118 0.029 0.176 0.088 0.118 0.118 0.118 0.029 0.059 0.029 0.029 0.000 0.000 0.000 0.029 0.000 34

CHTENG 0.000 0.000 0.000 0.000 0.000 0.000 0.100 0.250 0.100 0.100 0.050 0.200 0.100 0.000 0.000 0.050 0.000 0.050 0.000 0.000 20

Locus: 3

------------------

Pop Alleles Genes

----------------------------------------------------------------------------------------------------------------------------------------------------------------------------------------------------------------------------------------------------------------------------------------------------------------------------------------------------------------------------- -----

1 2 3 4 5 6 7 8 9 10 11 12 13 14 15 16 17 18 19 20 21 22 23 24 25 26 27 28 29 30 31 32 33 34 35 36 37 38 39 40 41 42 43 44 45 46 47 48 49 50 51 52 53 54 55 56 57 58 59 60 61

Ad2010 0.000 0.003 0.000 0.003 0.003 0.000 0.000 0.007 0.017 0.010 0.037 0.010 0.007 0.030 0.013 0.037 0.013 0.037 0.047 0.047 0.040 0.047 0.037 0.027 0.037 0.044 0.023 0.044 0.047 0.040 0.054 0.034 0.027 0.027 0.027 0.010 0.007 0.013 0.003 0.017 0.003 0.000 0.013 0.003 0.000 0.007 0.000 0.003 0.010 0.003 0.003 0.000 0.003 0.000 0.003 0.007 0.007 0.000 0.003 0.000 0.003 298

Ad2011 0.000 0.000 0.000 0.004 0.000 0.000 0.004 0.009 0.026 0.017 0.004 0.004 0.013 0.022 0.017 0.035 0.026 0.030 0.052 0.030 0.083 0.065 0.039 0.017 0.065 0.035 0.017 0.022 0.039 0.022 0.043 0.022 0.035 0.039 0.035 0.017 0.030 0.004 0.004 0.013 0.009 0.000 0.000 0.004 0.000 0.000 0.000 0.004 0.000 0.000 0.000 0.000 0.004 0.000 0.000 0.000 0.017 0.009 0.004 0.004 0.000 230

ad2012 0.004 0.004 0.000 0.017 0.000 0.008 0.004 0.017 0.021 0.000 0.033 0.013 0.017 0.017 0.008 0.054 0.021 0.025 0.046 0.042 0.042 0.050 0.054 0.021 0.008 0.071 0.037 0.021 0.058 0.033 0.025 0.025 0.021 0.042 0.013 0.025 0.008 0.017 0.000 0.000 0.013 0.008 0.004 0.004 0.000 0.000 0.004 0.004 0.000 0.000 0.004 0.017 0.004 0.000 0.000 0.000 0.000 0.008 0.000 0.000 0.008 240

LC 0.000 0.000 0.025 0.000 0.000 0.000 0.000 0.000 0.075 0.000 0.000 0.000 0.025 0.000 0.000 0.050 0.025 0.000 0.050 0.025 0.025 0.050 0.075 0.025 0.000 0.000 0.050 0.050 0.025 0.075 0.025 0.050 0.000 0.100 0.000 0.075 0.000 0.000 0.000 0.000 0.000 0.000 0.000 0.000 0.000 0.000 0.000 0.025 0.000 0.025 0.000 0.000 0.025 0.000 0.000 0.000 0.025 0.000 0.000 0.000 0.000 40

BT 0.000 0.000 0.000 0.000 0.000 0.000 0.000 0.029 0.029 0.000 0.000 0.059 0.000 0.000 0.000 0.029 0.029 0.029 0.088 0.000 0.000 0.000 0.000 0.059 0.029 0.059 0.000 0.000 0.059 0.059 0.029 0.029 0.029 0.088 0.000 0.000 0.059 0.000 0.059 0.000 0.000 0.000 0.029 0.000 0.000 0.000 0.000 0.029 0.000 0.000 0.000 0.000 0.000 0.000 0.000 0.029 0.000 0.000 0.029 0.029 0.000 34

Q 0.000 0.000 0.000 0.000 0.000 0.000 0.000 0.038 0.000 0.038 0.038 0.000 0.000 0.000 0.000 0.038 0.038 0.000 0.115 0.077 0.000 0.077 0.000 0.000 0.000 0.038 0.077 0.000 0.000 0.077 0.038 0.000 0.115 0.038 0.038 0.077 0.000 0.000 0.000 0.038 0.000 0.000 0.000 0.000 0.000 0.000 0.000 0.000 0.000 0.000 0.000 0.000 0.000 0.000 0.000 0.000 0.000 0.000 0.000 0.000 0.000 26

CHWENG 0.000 0.000 0.000 0.000 0.000 0.000 0.000 0.000 0.000 0.000 0.000 0.000 0.000 0.000 0.000 0.000 0.000 0.000 0.000 0.100 0.000 0.000 0.000 0.000 0.000 0.100 0.000 0.300 0.000 0.000 0.000 0.000 0.000 0.100 0.100 0.000 0.000 0.100 0.000 0.000 0.000 0.000 0.000 0.000 0.000 0.000 0.000 0.000 0.000 0.000 0.000 0.100 0.000 0.000 0.000 0.000 0.000 0.100 0.000 0.000 0.000 10

CHVILL 0.000 0.000 0.000 0.000 0.000 0.000 0.000 0.000 0.000 0.000 0.000 0.000 0.000 0.000 0.000 0.000 0.000 0.000 0.333 0.000 0.000 0.000 0.167 0.000 0.000 0.000 0.000 0.167 0.167 0.000 0.000 0.000 0.000 0.000 0.000 0.000 0.000 0.000 0.000 0.167 0.000 0.000 0.000 0.000 0.000 0.000 0.000 0.000 0.000 0.000 0.000 0.000 0.000 0.000 0.000 0.000 0.000 0.000 0.000 0.000 0.000 6

CHSWE 0.000 0.000 0.000 0.000 0.000 0.000 0.000 0.000 0.000 0.031 0.031 0.000 0.000 0.000 0.000 0.031 0.031 0.000 0.000 0.062 0.031 0.031 0.031 0.094 0.000 0.000 0.062 0.031 0.000 0.062 0.031 0.062 0.031 0.031 0.031 0.094 0.031 0.031 0.000 0.000 0.000 0.000 0.000 0.000 0.031 0.000 0.000 0.031 0.000 0.000 0.031 0.000 0.000 0.000 0.000 0.000 0.000 0.000 0.000 0.031 0.000 32

CHITA 0.000 0.000 0.000 0.000 0.000 0.000 0.000 0.000 0.000 0.000 0.000 0.000 0.000 0.000 0.000 0.125 0.000 0.000 0.125 0.000 0.125 0.125 0.000 0.000 0.000 0.125 0.000 0.000 0.000 0.000 0.000 0.125 0.000 0.000 0.000 0.000 0.000 0.000 0.000 0.000 0.000 0.000 0.125 0.000 0.000 0.000 0.000 0.000 0.000 0.000 0.000 0.000 0.125 0.000 0.000 0.000 0.000 0.000 0.000 0.000 0.000 8

CHBIRL 0.000 0.000 0.000 0.000 0.000 0.000 0.000 0.036 0.036 0.000 0.000 0.071 0.000 0.000 0.000 0.071 0.036 0.000 0.036 0.000 0.107 0.000 0.036 0.143 0.000 0.071 0.036 0.071 0.071 0.000 0.036 0.000 0.000 0.036 0.000 0.000 0.036 0.000 0.000 0.000 0.036 0.000 0.000 0.000 0.000 0.000 0.000 0.036 0.000 0.000 0.000 0.000 0.000 0.000 0.000 0.000 0.000 0.000 0.000 0.000 0.000 28

BNIRL 0.000 0.000 0.000 0.000 0.000 0.000 0.042 0.000 0.000 0.000 0.042 0.042 0.000 0.042 0.000 0.042 0.042 0.042 0.083 0.042 0.083 0.000 0.000 0.042 0.000 0.083 0.000 0.000 0.000 0.125 0.000 0.000 0.083 0.042 0.083 0.000 0.000 0.000 0.000 0.000 0.000 0.000 0.000 0.000 0.000 0.000 0.000 0.000 0.042 0.000 0.000 0.000 0.000 0.000 0.000 0.000 0.000 0.000 0.000 0.000 0.000 24

SPA 0.000 0.000 0.000 0.000 0.000 0.000 0.000 0.000 0.000 0.000 0.000 0.000 0.000 0.000 0.125 0.000 0.000 0.000 0.000 0.000 0.125 0.000 0.000 0.125 0.000 0.000 0.250 0.000 0.000 0.000 0.125 0.000 0.125 0.000 0.125 0.000 0.000 0.000 0.000 0.000 0.000 0.000 0.000 0.000 0.000 0.000 0.000 0.000 0.000 0.000 0.000 0.000 0.000 0.000 0.000 0.000 0.000 0.000 0.000 0.000 0.000 8

CNIRL 0.000 0.000 0.000 0.000 0.000 0.000 0.000 0.000 0.000 0.000 0.000 0.000 0.000 0.000 0.100 0.000 0.000 0.000 0.000 0.000 0.200 0.000 0.000 0.000 0.100 0.100 0.000 0.100 0.000 0.300 0.000 0.000 0.100 0.000 0.000 0.000 0.000 0.000 0.000 0.000 0.000 0.000 0.000 0.000 0.000 0.000 0.000 0.000 0.000 0.000 0.000 0.000 0.000 0.000 0.000 0.000 0.000 0.000 0.000 0.000 0.000 10

GER 0.000 0.000 0.000 0.000 0.000 0.000 0.000 0.000 0.000 0.000 0.000 0.000 0.000 0.000 0.100 0.200 0.000 0.100 0.000 0.000 0.000 0.000 0.000 0.000 0.000 0.100 0.100 0.000 0.000 0.000 0.000 0.000 0.100 0.000 0.100 0.000 0.000 0.100 0.000 0.000 0.000 0.100 0.000 0.000 0.000 0.000 0.000 0.000 0.000 0.000 0.000 0.000 0.000 0.000 0.000 0.000 0.000 0.000 0.000 0.000 0.000 10

BU 0.000 0.000 0.000 0.000 0.000 0.000 0.000 0.000 0.062 0.000 0.000 0.031 0.000 0.000 0.094 0.031 0.062 0.000 0.031 0.000 0.000 0.094 0.062 0.031 0.000 0.000 0.094 0.000 0.094 0.000 0.062 0.000 0.062 0.031 0.031 0.031 0.000 0.031 0.000 0.000 0.000 0.000 0.031 0.000 0.000 0.000 0.000 0.000 0.000 0.000 0.000 0.000 0.000 0.000 0.000 0.000 0.000 0.000 0.000 0.031 0.000 32

BL 0.000 0.000 0.000 0.000 0.000 0.000 0.000 0.000 0.000 0.045 0.000 0.000 0.000 0.045 0.045 0.045 0.000 0.045 0.045 0.000 0.091 0.091 0.000 0.000 0.045 0.045 0.000 0.045 0.045 0.000 0.000 0.000 0.045 0.045 0.000 0.136 0.000 0.045 0.000 0.000 0.000 0.000 0.000 0.000 0.000 0.000 0.000 0.000 0.000 0.000 0.045 0.045 0.000 0.000 0.000 0.000 0.000 0.000 0.000 0.000 0.000 22

SLC 0.000 0.000 0.000 0.000 0.000 0.000 0.143 0.000 0.000 0.000 0.000 0.000 0.000 0.071 0.071 0.000 0.071 0.071 0.000 0.000 0.143 0.000 0.000 0.000 0.071 0.000 0.071 0.000 0.000 0.071 0.000 0.000 0.000 0.071 0.071 0.000 0.000 0.000 0.000 0.000 0.000 0.000 0.000 0.000 0.000 0.000 0.000 0.000 0.000 0.000 0.000 0.000 0.000 0.000 0.000 0.000 0.000 0.000 0.071 0.000 0.000 14

LL 0.000 0.000 0.000 0.000 0.000 0.000 0.000 0.000 0.000 0.000 0.000 0.000 0.000 0.000 0.000 0.000 0.042 0.000 0.125 0.208 0.000 0.042 0.000 0.083 0.083 0.042 0.042 0.000 0.000 0.083 0.000 0.000 0.000 0.042 0.000 0.000 0.042 0.042 0.000 0.000 0.042 0.000 0.000 0.000 0.083 0.000 0.000 0.000 0.000 0.000 0.000 0.000 0.000 0.000 0.000 0.000 0.000 0.000 0.000 0.000 0.000 24

SLB 0.000 0.000 0.000 0.000 0.000 0.000 0.067 0.000 0.067 0.000 0.033 0.000 0.033 0.033 0.033 0.033 0.000 0.033 0.000 0.067 0.000 0.033 0.000 0.100 0.000 0.067 0.000 0.067 0.000 0.100 0.100 0.000 0.033 0.033 0.000 0.033 0.000 0.000 0.000 0.000 0.000 0.000 0.033 0.000 0.000 0.000 0.000 0.000 0.000 0.000 0.000 0.000 0.000 0.000 0.000 0.000 0.000 0.000 0.000 0.000 0.000 30

GL 0.000 0.000 0.000 0.000 0.000 0.000 0.000 0.038 0.038 0.000 0.000 0.000 0.000 0.000 0.000 0.000 0.038 0.000 0.038 0.000 0.038 0.115 0.115 0.038 0.115 0.077 0.038 0.038 0.000 0.077 0.038 0.000 0.000 0.077 0.000 0.038 0.000 0.000 0.038 0.000 0.000 0.000 0.000 0.000 0.000 0.000 0.000 0.000 0.000 0.000 0.000 0.000 0.000 0.000 0.000 0.000 0.000 0.000 0.000 0.000 0.000 26

DK 0.000 0.000 0.000 0.000 0.000 0.000 0.000 0.028 0.000 0.000 0.000 0.000 0.000 0.000 0.056 0.028 0.000 0.056 0.056 0.000 0.028 0.111 0.000 0.056 0.028 0.083 0.083 0.111 0.028 0.000 0.000 0.028 0.028 0.028 0.000 0.000 0.000 0.000 0.000 0.056 0.000 0.000 0.028 0.000 0.000 0.000 0.000 0.000 0.000 0.000 0.000 0.000 0.000 0.000 0.000 0.000 0.056 0.028 0.000 0.000 0.000 36

FI 0.000 0.000 0.000 0.000 0.000 0.025 0.000 0.075 0.025 0.000 0.000 0.100 0.025 0.000 0.000 0.050 0.075 0.000 0.000 0.000 0.100 0.025 0.075 0.025 0.025 0.000 0.025 0.025 0.025 0.025 0.025 0.000 0.025 0.000 0.000 0.025 0.025 0.000 0.000 0.025 0.000 0.000 0.000 0.000 0.000 0.000 0.000 0.000 0.000 0.000 0.000 0.000 0.000 0.050 0.000 0.000 0.025 0.025 0.025 0.000 0.025 40

PT 0.000 0.000 0.000 0.000 0.000 0.000 0.000 0.088 0.000 0.000 0.000 0.000 0.029 0.000 0.000 0.059 0.000 0.000 0.029 0.000 0.029 0.088 0.000 0.059 0.000 0.059 0.088 0.059 0.088 0.088 0.059 0.029 0.029 0.029 0.059 0.000 0.000 0.000 0.000 0.000 0.000 0.000 0.000 0.000 0.029 0.000 0.000 0.000 0.000 0.000 0.000 0.000 0.000 0.000 0.000 0.000 0.000 0.000 0.000 0.000 0.000 34

WK 0.000 0.000 0.000 0.029 0.000 0.000 0.000 0.029 0.000 0.000 0.029 0.059 0.000 0.000 0.000 0.000 0.088 0.029 0.000 0.029 0.059 0.000 0.059 0.029 0.000 0.029 0.000 0.059 0.088 0.029 0.118 0.029 0.000 0.088 0.000 0.000 0.029 0.000 0.000 0.000 0.029 0.059 0.000 0.000 0.000 0.000 0.000 0.000 0.000 0.000 0.000 0.000 0.000 0.000 0.000 0.000 0.000 0.000 0.000 0.000 0.000 34

CHTENG 0.000 0.000 0.000 0.000 0.000 0.000 0.000 0.000 0.050 0.000 0.050 0.050 0.000 0.000 0.000 0.100 0.000 0.050 0.000 0.000 0.200 0.000 0.050 0.000 0.050 0.050 0.050 0.000 0.000 0.000 0.000 0.000 0.000 0.050 0.100 0.100 0.000 0.000 0.000 0.000 0.000 0.000 0.000 0.000 0.000 0.000 0.000 0.000 0.050 0.000 0.000 0.000 0.000 0.000 0.000 0.000 0.000 0.000 0.000 0.000 0.000 20

Locus: 4

------------------

Pop Alleles Genes

----------------------------------------------------------------------------------------------------------------------------------------------------------------------------------------- -----

1 2 3 4 5 6 7 8 9 10 11 12 13 14 15 16 17 18 19 20 21 22 23 24 25 26 27 28 29 30 31

Ad2010 0.000 0.000 0.000 0.000 0.087 0.000 0.215 0.376 0.077 0.114 0.010 0.000 0.003 0.003 0.007 0.000 0.010 0.003 0.007 0.003 0.000 0.000 0.003 0.000 0.000 0.000 0.013 0.003 0.044 0.020 0.000 298

Ad2011 0.000 0.000 0.004 0.000 0.044 0.004 0.189 0.386 0.075 0.154 0.009 0.000 0.000 0.000 0.004 0.009 0.009 0.004 0.000 0.009 0.000 0.004 0.004 0.000 0.004 0.000 0.009 0.004 0.061 0.009 0.004 228

ad2012 0.000 0.004 0.000 0.000 0.042 0.004 0.140 0.445 0.076 0.093 0.013 0.004 0.004 0.000 0.000 0.000 0.013 0.004 0.000 0.004 0.004 0.000 0.021 0.000 0.000 0.004 0.013 0.017 0.076 0.017 0.000 236

LC 0.050 0.000 0.000 0.000 0.150 0.000 0.125 0.325 0.000 0.200 0.025 0.000 0.000 0.000 0.025 0.000 0.000 0.000 0.000 0.000 0.000 0.000 0.000 0.050 0.000 0.000 0.000 0.025 0.025 0.000 0.000 40

BT 0.000 0.000 0.000 0.000 0.029 0.029 0.000 0.618 0.088 0.206 0.000 0.000 0.000 0.000 0.000 0.000 0.000 0.000 0.000 0.000 0.000 0.000 0.000 0.000 0.000 0.000 0.000 0.000 0.000 0.029 0.000 34

Q 0.000 0.000 0.000 0.000 0.038 0.000 0.346 0.308 0.231 0.038 0.000 0.000 0.000 0.000 0.000 0.000 0.000 0.000 0.000 0.000 0.000 0.038 0.000 0.000 0.000 0.000 0.000 0.000 0.000 0.000 0.000 26

CHWENG 0.000 0.000 0.000 0.000 0.100 0.000 0.200 0.400 0.000 0.300 0.000 0.000 0.000 0.000 0.000 0.000 0.000 0.000 0.000 0.000 0.000 0.000 0.000 0.000 0.000 0.000 0.000 0.000 0.000 0.000 0.000 10

CHVILL 0.000 0.000 0.000 0.000 0.167 0.000 0.167 0.333 0.000 0.000 0.000 0.000 0.000 0.000 0.000 0.000 0.000 0.000 0.000 0.000 0.000 0.000 0.167 0.000 0.000 0.000 0.167 0.000 0.000 0.000 0.000 6

CHSWE 0.000 0.000 0.000 0.000 0.062 0.000 0.156 0.344 0.062 0.156 0.000 0.000 0.031 0.000 0.000 0.000 0.031 0.000 0.000 0.000 0.000 0.000 0.062 0.000 0.000 0.000 0.000 0.031 0.062 0.000 0.000 32

CHITA 0.000 0.000 0.000 0.000 0.000 0.000 0.250 0.375 0.125 0.125 0.000 0.000 0.000 0.000 0.000 0.000 0.125 0.000 0.000 0.000 0.000 0.000 0.000 0.000 0.000 0.000 0.000 0.000 0.000 0.000 0.000 8

CHBIRL 0.071 0.000 0.000 0.000 0.107 0.000 0.179 0.357 0.000 0.071 0.000 0.000 0.000 0.000 0.000 0.000 0.000 0.000 0.000 0.000 0.000 0.000 0.000 0.000 0.000 0.000 0.000 0.071 0.107 0.036 0.000 28

BNIRL 0.000 0.000 0.000 0.000 0.042 0.000 0.125 0.583 0.000 0.125 0.000 0.000 0.000 0.000 0.000 0.000 0.000 0.000 0.042 0.000 0.042 0.000 0.000 0.000 0.000 0.000 0.000 0.000 0.000 0.042 0.000 24

SPA 0.000 0.000 0.000 0.000 0.000 0.000 0.125 0.500 0.125 0.125 0.000 0.000 0.000 0.125 0.000 0.000 0.000 0.000 0.000 0.000 0.000 0.000 0.000 0.000 0.000 0.000 0.000 0.000 0.000 0.000 0.000 8

CNIRL 0.000 0.000 0.000 0.000 0.000 0.000 0.100 0.500 0.000 0.400 0.000 0.000 0.000 0.000 0.000 0.000 0.000 0.000 0.000 0.000 0.000 0.000 0.000 0.000 0.000 0.000 0.000 0.000 0.000 0.000 0.000 10

GER 0.100 0.000 0.000 0.000 0.000 0.000 0.100 0.500 0.000 0.200 0.000 0.000 0.000 0.000 0.000 0.000 0.000 0.000 0.000 0.000 0.000 0.000 0.000 0.000 0.000 0.000 0.000 0.000 0.100 0.000 0.000 10

BU 0.000 0.000 0.000 0.000 0.219 0.000 0.281 0.375 0.062 0.000 0.000 0.000 0.000 0.000 0.000 0.000 0.000 0.000 0.000 0.000 0.000 0.000 0.000 0.000 0.000 0.000 0.000 0.000 0.062 0.000 0.000 32

BL 0.000 0.000 0.000 0.000 0.182 0.000 0.136 0.227 0.409 0.000 0.000 0.000 0.000 0.000 0.045 0.000 0.000 0.000 0.000 0.000 0.000 0.000 0.000 0.000 0.000 0.000 0.000 0.000 0.000 0.000 0.000 22

SLC 0.000 0.000 0.000 0.000 0.143 0.000 0.071 0.500 0.143 0.071 0.000 0.000 0.000 0.000 0.000 0.000 0.000 0.000 0.000 0.000 0.000 0.000 0.000 0.000 0.000 0.000 0.000 0.000 0.000 0.071 0.000 14

LL 0.038 0.000 0.000 0.000 0.038 0.000 0.192 0.346 0.231 0.115 0.000 0.038 0.000 0.000 0.000 0.000 0.000 0.000 0.000 0.000 0.000 0.000 0.000 0.000 0.000 0.000 0.000 0.000 0.000 0.000 0.000 26

SLB 0.000 0.000 0.000 0.000 0.100 0.000 0.133 0.400 0.167 0.067 0.033 0.000 0.000 0.000 0.033 0.000 0.000 0.000 0.000 0.000 0.000 0.000 0.000 0.000 0.000 0.000 0.033 0.000 0.033 0.000 0.000 30

GL 0.000 0.000 0.000 0.000 0.192 0.000 0.077 0.385 0.077 0.192 0.000 0.000 0.000 0.000 0.038 0.000 0.000 0.000 0.000 0.000 0.000 0.000 0.000 0.000 0.000 0.000 0.000 0.000 0.038 0.000 0.000 26

DK 0.000 0.000 0.000 0.000 0.000 0.053 0.105 0.526 0.053 0.105 0.000 0.000 0.000 0.000 0.000 0.000 0.000 0.000 0.000 0.000 0.000 0.000 0.026 0.000 0.000 0.000 0.000 0.026 0.105 0.000 0.000 38

FI 0.000 0.000 0.000 0.025 0.000 0.000 0.150 0.400 0.025 0.200 0.075 0.000 0.000 0.000 0.000 0.025 0.025 0.000 0.000 0.000 0.000 0.000 0.000 0.000 0.000 0.000 0.000 0.000 0.075 0.000 0.000 40

PT 0.000 0.000 0.000 0.000 0.029 0.000 0.147 0.529 0.059 0.088 0.000 0.000 0.000 0.000 0.029 0.000 0.000 0.000 0.000 0.000 0.000 0.000 0.000 0.000 0.000 0.000 0.000 0.000 0.088 0.029 0.000 34

WK 0.000 0.000 0.000 0.000 0.029 0.029 0.206 0.382 0.000 0.265 0.000 0.000 0.000 0.000 0.000 0.000 0.000 0.000 0.000 0.000 0.000 0.000 0.000 0.000 0.000 0.029 0.000 0.029 0.029 0.000 0.000 34

CHTENG 0.000 0.000 0.000 0.000 0.050 0.000 0.050 0.450 0.100 0.250 0.000 0.000 0.000 0.000 0.050 0.000 0.000 0.000 0.000 0.000 0.000 0.000 0.000 0.000 0.000 0.000 0.000 0.000 0.050 0.000 0.000 20

Locus: 5

------------------

Pop Alleles Genes

----------------------------------------------------------------------------------------------------------------------------- -----

1 2 3 4 5 6 7 8 9 10 11 12 13 14 15 16 17 18 19 20 21

Ad2010 0.000 0.000 0.007 0.023 0.017 0.023 0.076 0.348 0.129 0.119 0.126 0.033 0.036 0.017 0.010 0.000 0.020 0.007 0.000 0.010 0.000 302

Ad2011 0.004 0.004 0.000 0.030 0.022 0.030 0.043 0.365 0.117 0.139 0.117 0.030 0.061 0.022 0.004 0.004 0.004 0.000 0.000 0.000 0.000 230

ad2012 0.000 0.000 0.004 0.025 0.033 0.029 0.067 0.321 0.150 0.117 0.096 0.054 0.050 0.013 0.013 0.004 0.017 0.000 0.000 0.008 0.000 240

LC 0.000 0.000 0.000 0.000 0.050 0.050 0.025 0.350 0.125 0.150 0.075 0.025 0.025 0.075 0.025 0.000 0.000 0.000 0.025 0.000 0.000 40

BT 0.000 0.000 0.000 0.000 0.088 0.029 0.000 0.471 0.147 0.088 0.029 0.029 0.029 0.088 0.000 0.000 0.000 0.000 0.000 0.000 0.000 34

Q 0.000 0.000 0.000 0.038 0.038 0.000 0.000 0.346 0.231 0.115 0.115 0.038 0.038 0.000 0.000 0.000 0.000 0.000 0.038 0.000 0.000 26

CHWENG 0.000 0.000 0.000 0.000 0.000 0.000 0.000 0.700 0.200 0.000 0.100 0.000 0.000 0.000 0.000 0.000 0.000 0.000 0.000 0.000 0.000 10

CHVILL 0.000 0.000 0.000 0.000 0.000 0.000 0.167 0.333 0.000 0.333 0.167 0.000 0.000 0.000 0.000 0.000 0.000 0.000 0.000 0.000 0.000 6

CHSWE 0.000 0.000 0.000 0.000 0.000 0.000 0.031 0.219 0.219 0.250 0.062 0.094 0.094 0.000 0.000 0.000 0.000 0.031 0.000 0.000 0.000 32

CHITA 0.000 0.000 0.000 0.000 0.000 0.000 0.125 0.250 0.000 0.125 0.250 0.125 0.000 0.000 0.125 0.000 0.000 0.000 0.000 0.000 0.000 8

CHBIRL 0.000 0.000 0.000 0.000 0.000 0.071 0.036 0.357 0.143 0.107 0.107 0.107 0.071 0.000 0.000 0.000 0.000 0.000 0.000 0.000 0.000 28

BNIRL 0.000 0.000 0.000 0.042 0.042 0.083 0.125 0.292 0.042 0.083 0.125 0.000 0.042 0.000 0.042 0.000 0.000 0.000 0.042 0.000 0.042 24

SPA 0.000 0.000 0.000 0.000 0.000 0.000 0.000 0.250 0.250 0.000 0.125 0.125 0.000 0.000 0.000 0.000 0.250 0.000 0.000 0.000 0.000 8

CNIRL 0.000 0.000 0.000 0.100 0.000 0.000 0.000 0.100 0.400 0.200 0.100 0.100 0.000 0.000 0.000 0.000 0.000 0.000 0.000 0.000 0.000 10

GER 0.000 0.000 0.000 0.000 0.000 0.000 0.100 0.200 0.100 0.300 0.300 0.000 0.000 0.000 0.000 0.000 0.000 0.000 0.000 0.000 0.000 10

BU 0.000 0.000 0.000 0.031 0.000 0.156 0.094 0.344 0.094 0.094 0.031 0.062 0.062 0.000 0.031 0.000 0.000 0.000 0.000 0.000 0.000 32

BL 0.000 0.000 0.000 0.045 0.091 0.045 0.000 0.318 0.182 0.227 0.045 0.000 0.045 0.000 0.000 0.000 0.000 0.000 0.000 0.000 0.000 22

SLC 0.000 0.000 0.000 0.000 0.000 0.000 0.000 0.429 0.214 0.214 0.071 0.071 0.000 0.000 0.000 0.000 0.000 0.000 0.000 0.000 0.000 14

LL 0.000 0.000 0.000 0.038 0.000 0.038 0.038 0.423 0.077 0.038 0.154 0.115 0.077 0.000 0.000 0.000 0.000 0.000 0.000 0.000 0.000 26

SLB 0.000 0.000 0.000 0.000 0.000 0.067 0.033 0.267 0.167 0.133 0.067 0.067 0.133 0.000 0.067 0.000 0.000 0.000 0.000 0.000 0.000 30

GL 0.000 0.000 0.000 0.000 0.038 0.038 0.077 0.346 0.192 0.115 0.115 0.000 0.038 0.000 0.000 0.000 0.000 0.000 0.038 0.000 0.000 26

DK 0.000 0.000 0.000 0.026 0.000 0.026 0.026 0.263 0.158 0.105 0.079 0.053 0.132 0.000 0.000 0.053 0.079 0.000 0.000 0.000 0.000 38

FI 0.000 0.000 0.000 0.075 0.000 0.025 0.125 0.275 0.175 0.125 0.125 0.000 0.050 0.025 0.000 0.000 0.000 0.000 0.000 0.000 0.000 40

PT 0.000 0.000 0.000 0.000 0.000 0.000 0.088 0.382 0.176 0.118 0.118 0.000 0.059 0.000 0.000 0.029 0.029 0.000 0.000 0.000 0.000 34

WK 0.029 0.000 0.000 0.029 0.000 0.000 0.147 0.324 0.059 0.088 0.029 0.088 0.088 0.029 0.088 0.000 0.000 0.000 0.000 0.000 0.000 34

CHTENG 0.000 0.000 0.000 0.050 0.000 0.000 0.100 0.300 0.100 0.250 0.000 0.050 0.000 0.050 0.050 0.000 0.050 0.000 0.000 0.000 0.000 20

Locus: 6

------------------

Pop Alleles Genes

----------------------------------------- -----

1 2 3 4 5 6 7

Ad2010 0.000 0.040 0.010 0.007 0.943 0.000 0.000 300

Ad2011 0.000 0.065 0.000 0.004 0.904 0.026 0.000 230

ad2012 0.000 0.058 0.008 0.012 0.905 0.004 0.012 242

LC 0.000 0.025 0.000 0.050 0.925 0.000 0.000 40

BT 0.000 0.029 0.029 0.029 0.912 0.000 0.000 34

Q 0.000 0.038 0.000 0.000 0.923 0.038 0.000 26

CHWENG 0.000 0.100 0.000 0.000 0.900 0.000 0.000 10

CHVILL 0.000 0.000 0.167 0.000 0.833 0.000 0.000 6

CHSWE 0.000 0.156 0.000 0.000 0.844 0.000 0.000 32

CHITA 0.000 0.000 0.000 0.000 1.000 0.000 0.000 8

CHBIRL 0.000 0.036 0.000 0.000 0.964 0.000 0.000 28

BNIRL 0.000 0.083 0.000 0.000 0.917 0.000 0.000 24

SPA 0.000 0.000 0.000 0.000 1.000 0.000 0.000 8

CNIRL 0.000 0.000 0.000 0.100 0.900 0.000 0.000 10

GER 0.000 0.100 0.000 0.000 0.900 0.000 0.000 10

BU 0.000 0.094 0.031 0.031 0.844 0.000 0.000 32

BL 0.000 0.000 0.000 0.000 1.000 0.000 0.000 22

SLC 0.000 0.000 0.071 0.000 0.929 0.000 0.000 14

LL 0.000 0.038 0.000 0.000 0.962 0.000 0.000 26

SLB 0.000 0.067 0.033 0.000 0.900 0.000 0.000 30

GL 0.000 0.115 0.000 0.038 0.808 0.038 0.000 26

DK 0.026 0.132 0.000 0.000 0.816 0.000 0.026 38

FI 0.000 0.025 0.000 0.025 0.950 0.000 0.000 40

PT 0.000 0.029 0.000 0.000 0.971 0.000 0.000 34

WK 0.000 0.000 0.000 0.059 0.941 0.000 0.000 34

CHTENG 0.000 0.050 0.000 0.000 0.950 0.000 0.000 20

Locus: 7

------------------

Pop Alleles Genes

----------------------------------------------------------------------------------------------------------------------------------- -----

1 2 3 4 5 6 7 8 9 10 11 12 13 14 15 16 17 18 19 20 21 22

Ad2010 0.000 0.000 0.007 0.023 0.000 0.026 0.003 0.010 0.013 0.106 0.060 0.030 0.285 0.179 0.149 0.033 0.050 0.020 0.007 0.000 0.000 0.000 302

Ad2011 0.000 0.000 0.000 0.013 0.004 0.009 0.013 0.022 0.022 0.057 0.092 0.026 0.272 0.180 0.158 0.053 0.039 0.022 0.009 0.000 0.004 0.004 228

ad2012 0.004 0.000 0.008 0.013 0.004 0.000 0.021 0.008 0.025 0.121 0.087 0.054 0.242 0.196 0.121 0.025 0.046 0.008 0.004 0.008 0.000 0.004 240

LC 0.000 0.026 0.000 0.026 0.000 0.000 0.000 0.026 0.000 0.026 0.026 0.026 0.237 0.184 0.158 0.079 0.158 0.026 0.000 0.000 0.000 0.000 38

BT 0.000 0.000 0.000 0.000 0.000 0.000 0.000 0.029 0.000 0.088 0.088 0.059 0.324 0.147 0.147 0.000 0.029 0.029 0.059 0.000 0.000 0.000 34

Q 0.000 0.000 0.000 0.000 0.000 0.038 0.038 0.000 0.000 0.115 0.077 0.038 0.269 0.192 0.154 0.038 0.038 0.000 0.000 0.000 0.000 0.000 26

CHWENG 0.000 0.000 0.000 0.000 0.000 0.000 0.000 0.000 0.000 0.000 0.200 0.000 0.100 0.400 0.100 0.000 0.100 0.000 0.000 0.100 0.000 0.000 10

CHVILL 0.000 0.000 0.000 0.000 0.000 0.000 0.167 0.000 0.000 0.333 0.000 0.000 0.333 0.000 0.167 0.000 0.000 0.000 0.000 0.000 0.000 0.000 6

CHSWE 0.000 0.000 0.000 0.000 0.000 0.031 0.031 0.000 0.000 0.094 0.062 0.094 0.250 0.281 0.031 0.094 0.031 0.000 0.000 0.000 0.000 0.000 32

CHITA 0.000 0.000 0.000 0.125 0.000 0.000 0.000 0.000 0.000 0.125 0.250 0.250 0.125 0.125 0.000 0.000 0.000 0.000 0.000 0.000 0.000 0.000 8

CHBIRL 0.000 0.000 0.000 0.107 0.000 0.000 0.000 0.000 0.000 0.036 0.107 0.000 0.321 0.286 0.036 0.000 0.107 0.000 0.000 0.000 0.000 0.000 28

BNIRL 0.000 0.000 0.000 0.000 0.000 0.000 0.000 0.000 0.083 0.083 0.042 0.042 0.375 0.167 0.167 0.000 0.000 0.000 0.000 0.042 0.000 0.000 24

SPA 0.000 0.000 0.000 0.000 0.000 0.000 0.000 0.000 0.000 0.125 0.000 0.000 0.125 0.000 0.625 0.000 0.000 0.000 0.125 0.000 0.000 0.000 8

CNIRL 0.000 0.000 0.000 0.000 0.000 0.000 0.000 0.000 0.000 0.100 0.000 0.100 0.500 0.000 0.300 0.000 0.000 0.000 0.000 0.000 0.000 0.000 10

GER 0.000 0.000 0.000 0.000 0.000 0.100 0.000 0.000 0.000 0.200 0.100 0.000 0.100 0.100 0.300 0.000 0.100 0.000 0.000 0.000 0.000 0.000 10

BU 0.000 0.000 0.000 0.000 0.000 0.031 0.000 0.000 0.062 0.156 0.062 0.000 0.281 0.094 0.188 0.031 0.031 0.000 0.062 0.000 0.000 0.000 32

BL 0.000 0.000 0.000 0.000 0.000 0.000 0.000 0.000 0.000 0.100 0.000 0.000 0.250 0.400 0.200 0.050 0.000 0.000 0.000 0.000 0.000 0.000 20

SLC 0.000 0.000 0.000 0.000 0.000 0.000 0.071 0.000 0.000 0.000 0.000 0.000 0.500 0.286 0.071 0.000 0.071 0.000 0.000 0.000 0.000 0.000 14

LL 0.000 0.000 0.000 0.038 0.000 0.000 0.000 0.000 0.000 0.115 0.154 0.000 0.269 0.231 0.038 0.000 0.038 0.038 0.038 0.038 0.000 0.000 26

SLB 0.000 0.000 0.033 0.033 0.000 0.033 0.033 0.000 0.000 0.033 0.067 0.033 0.200 0.367 0.067 0.033 0.067 0.000 0.000 0.000 0.000 0.000 30

GL 0.000 0.000 0.038 0.000 0.000 0.000 0.000 0.000 0.115 0.000 0.115 0.000 0.308 0.154 0.154 0.038 0.077 0.000 0.000 0.000 0.000 0.000 26

DK 0.000 0.000 0.000 0.000 0.000 0.000 0.000 0.000 0.026 0.079 0.026 0.000 0.263 0.237 0.211 0.105 0.026 0.026 0.000 0.000 0.000 0.000 38

FI 0.000 0.000 0.025 0.000 0.000 0.000 0.000 0.000 0.050 0.150 0.000 0.100 0.200 0.300 0.150 0.000 0.025 0.000 0.000 0.000 0.000 0.000 40

PT 0.000 0.000 0.062 0.000 0.031 0.000 0.031 0.000 0.000 0.125 0.125 0.000 0.281 0.156 0.125 0.031 0.031 0.000 0.000 0.000 0.000 0.000 32

WK 0.000 0.000 0.000 0.000 0.000 0.000 0.000 0.000 0.000 0.094 0.062 0.000 0.281 0.312 0.094 0.062 0.000 0.062 0.031 0.000 0.000 0.000 32

CHTENG 0.000 0.000 0.000 0.000 0.000 0.050 0.000 0.000 0.000 0.350 0.050 0.000 0.200 0.200 0.000 0.000 0.000 0.000 0.050 0.100 0.000 0.000 20

Locus: 8

------------------

Pop Alleles Genes

----------------------------------------------------------------------------------------------------------------------------- -----

1 2 3 4 5 6 7 8 9 10 11 12 13 14 15 16 17 18 19 20 21

Ad2010 0.000 0.003 0.007 0.000 0.030 0.188 0.057 0.060 0.097 0.235 0.205 0.037 0.017 0.003 0.013 0.000 0.023 0.017 0.003 0.003 0.000 298

Ad2011 0.004 0.000 0.004 0.000 0.026 0.145 0.048 0.018 0.079 0.206 0.219 0.105 0.026 0.022 0.013 0.009 0.031 0.022 0.009 0.009 0.004 228

ad2012 0.000 0.004 0.008 0.008 0.008 0.161 0.025 0.081 0.119 0.301 0.148 0.034 0.025 0.013 0.004 0.021 0.017 0.008 0.013 0.000 0.000 236

LC 0.000 0.000 0.000 0.000 0.053 0.263 0.000 0.132 0.026 0.184 0.184 0.053 0.000 0.000 0.000 0.026 0.026 0.053 0.000 0.000 0.000 38

BT 0.000 0.000 0.000 0.000 0.000 0.147 0.088 0.029 0.088 0.147 0.206 0.176 0.029 0.000 0.000 0.000 0.088 0.000 0.000 0.000 0.000 34

Q 0.000 0.045 0.000 0.000 0.000 0.182 0.091 0.136 0.091 0.091 0.182 0.045 0.000 0.000 0.045 0.000 0.091 0.000 0.000 0.000 0.000 22

CHWENG 0.000 0.000 0.000 0.000 0.000 0.300 0.000 0.000 0.000 0.200 0.200 0.000 0.100 0.100 0.100 0.000 0.000 0.000 0.000 0.000 0.000 10

CHVILL 0.000 0.000 0.000 0.000 0.000 0.167 0.000 0.000 0.000 0.167 0.500 0.000 0.000 0.000 0.000 0.000 0.167 0.000 0.000 0.000 0.000 6

CHSWE 0.000 0.000 0.000 0.000 0.000 0.062 0.156 0.094 0.156 0.094 0.250 0.062 0.031 0.000 0.000 0.000 0.031 0.062 0.000 0.000 0.000 32

CHITA 0.000 0.000 0.000 0.000 0.125 0.000 0.125 0.000 0.125 0.125 0.125 0.125 0.000 0.000 0.000 0.000 0.250 0.000 0.000 0.000 0.000 8

CHBIRL 0.000 0.000 0.000 0.000 0.000 0.250 0.000 0.000 0.107 0.250 0.250 0.107 0.000 0.000 0.036 0.000 0.000 0.000 0.000 0.000 0.000 28

BNIRL 0.000 0.000 0.000 0.042 0.042 0.250 0.000 0.167 0.000 0.208 0.167 0.083 0.000 0.042 0.000 0.000 0.000 0.000 0.000 0.000 0.000 24

SPA 0.000 0.000 0.000 0.000 0.000 0.125 0.000 0.000 0.000 0.250 0.250 0.125 0.000 0.000 0.250 0.000 0.000 0.000 0.000 0.000 0.000 8

CNIRL 0.000 0.000 0.000 0.000 0.100 0.500 0.000 0.000 0.000 0.000 0.300 0.100 0.000 0.000 0.000 0.000 0.000 0.000 0.000 0.000 0.000 10

GER 0.000 0.000 0.000 0.000 0.000 0.000 0.000 0.200 0.000 0.300 0.300 0.200 0.000 0.000 0.000 0.000 0.000 0.000 0.000 0.000 0.000 10

BU 0.000 0.000 0.000 0.031 0.000 0.188 0.031 0.000 0.094 0.188 0.375 0.000 0.000 0.000 0.062 0.000 0.031 0.000 0.000 0.000 0.000 32

BL 0.000 0.000 0.000 0.000 0.000 0.150 0.000 0.000 0.100 0.300 0.250 0.050 0.100 0.000 0.000 0.000 0.050 0.000 0.000 0.000 0.000 20

SLC 0.000 0.000 0.000 0.000 0.071 0.143 0.071 0.000 0.071 0.071 0.286 0.214 0.071 0.000 0.000 0.000 0.000 0.000 0.000 0.000 0.000 14

LL 0.000 0.000 0.000 0.000 0.042 0.333 0.000 0.083 0.042 0.000 0.375 0.042 0.042 0.000 0.000 0.000 0.042 0.000 0.000 0.000 0.000 24

SLB 0.000 0.000 0.000 0.000 0.000 0.179 0.071 0.036 0.036 0.214 0.321 0.036 0.036 0.000 0.000 0.000 0.036 0.036 0.000 0.000 0.000 28

GL 0.000 0.000 0.000 0.083 0.000 0.208 0.042 0.000 0.208 0.083 0.167 0.000 0.000 0.042 0.000 0.000 0.083 0.042 0.000 0.000 0.042 24

DK 0.000 0.000 0.000 0.000 0.053 0.289 0.000 0.053 0.026 0.237 0.263 0.000 0.000 0.000 0.026 0.026 0.026 0.000 0.000 0.000 0.000 38

FI 0.000 0.000 0.000 0.000 0.025 0.175 0.000 0.050 0.225 0.150 0.200 0.125 0.025 0.000 0.000 0.000 0.000 0.025 0.000 0.000 0.000 40

PT 0.000 0.036 0.000 0.000 0.000 0.214 0.000 0.071 0.071 0.143 0.214 0.071 0.000 0.000 0.071 0.036 0.036 0.000 0.036 0.000 0.000 28

WK 0.000 0.000 0.000 0.000 0.059 0.118 0.029 0.000 0.118 0.294 0.235 0.059 0.029 0.029 0.000 0.000 0.029 0.000 0.000 0.000 0.000 34

CHTENG 0.000 0.000 0.000 0.000 0.050 0.200 0.050 0.000 0.100 0.100 0.350 0.050 0.000 0.000 0.000 0.000 0.050 0.000 0.000 0.050 0.000 20

Locus: 9

------------------

Pop Alleles Genes

----------------------------------------------------------------------------------------------------------------------------------- -----

1 2 3 4 5 6 7 8 9 10 11 12 13 14 15 16 17 18 19 20 21 22

Ad2010 0.007 0.010 0.003 0.023 0.093 0.477 0.073 0.142 0.073 0.023 0.026 0.007 0.000 0.007 0.010 0.003 0.007 0.010 0.007 0.000 0.000 0.000 302

Ad2011 0.000 0.004 0.009 0.035 0.061 0.465 0.092 0.180 0.075 0.026 0.022 0.009 0.004 0.000 0.004 0.004 0.004 0.004 0.000 0.000 0.000 0.000 228

ad2012 0.004 0.000 0.013 0.021 0.079 0.458 0.087 0.158 0.083 0.029 0.025 0.013 0.004 0.000 0.004 0.004 0.000 0.004 0.000 0.004 0.004 0.004 240

LC 0.000 0.000 0.000 0.026 0.158 0.395 0.158 0.158 0.053 0.000 0.000 0.026 0.000 0.000 0.000 0.000 0.000 0.026 0.000 0.000 0.000 0.000 38

BT 0.000 0.000 0.000 0.000 0.029 0.412 0.088 0.265 0.147 0.059 0.000 0.000 0.000 0.000 0.000 0.000 0.000 0.000 0.000 0.000 0.000 0.000 34

Q 0.000 0.000 0.000 0.038 0.038 0.654 0.000 0.154 0.077 0.000 0.000 0.000 0.000 0.038 0.000 0.000 0.000 0.000 0.000 0.000 0.000 0.000 26

CHWENG 0.000 0.000 0.000 0.000 0.000 0.600 0.100 0.200 0.100 0.000 0.000 0.000 0.000 0.000 0.000 0.000 0.000 0.000 0.000 0.000 0.000 0.000 10

CHVILL 0.000 0.000 0.000 0.000 0.000 0.500 0.167 0.167 0.167 0.000 0.000 0.000 0.000 0.000 0.000 0.000 0.000 0.000 0.000 0.000 0.000 0.000 6

CHSWE 0.000 0.000 0.000 0.031 0.156 0.375 0.062 0.219 0.031 0.062 0.031 0.000 0.000 0.000 0.000 0.031 0.000 0.000 0.000 0.000 0.000 0.000 32

CHITA 0.000 0.000 0.000 0.125 0.000 0.375 0.125 0.125 0.250 0.000 0.000 0.000 0.000 0.000 0.000 0.000 0.000 0.000 0.000 0.000 0.000 0.000 8

CHBIRL 0.000 0.000 0.000 0.071 0.000 0.643 0.000 0.143 0.071 0.036 0.036 0.000 0.000 0.000 0.000 0.000 0.000 0.000 0.000 0.000 0.000 0.000 28

BNIRL 0.000 0.000 0.000 0.042 0.083 0.500 0.167 0.042 0.083 0.000 0.042 0.000 0.000 0.000 0.000 0.000 0.042 0.000 0.000 0.000 0.000 0.000 24

SPA 0.000 0.000 0.000 0.125 0.000 0.625 0.000 0.000 0.250 0.000 0.000 0.000 0.000 0.000 0.000 0.000 0.000 0.000 0.000 0.000 0.000 0.000 8

CNIRL 0.000 0.000 0.000 0.000 0.000 0.400 0.100 0.300 0.100 0.100 0.000 0.000 0.000 0.000 0.000 0.000 0.000 0.000 0.000 0.000 0.000 0.000 10

GER 0.000 0.000 0.000 0.000 0.000 0.300 0.200 0.200 0.000 0.300 0.000 0.000 0.000 0.000 0.000 0.000 0.000 0.000 0.000 0.000 0.000 0.000 10

BU 0.000 0.031 0.000 0.031 0.062 0.406 0.062 0.125 0.062 0.094 0.000 0.031 0.000 0.000 0.062 0.000 0.031 0.000 0.000 0.000 0.000 0.000 32

BL 0.000 0.000 0.000 0.000 0.100 0.650 0.000 0.150 0.100 0.000 0.000 0.000 0.000 0.000 0.000 0.000 0.000 0.000 0.000 0.000 0.000 0.000 20

SLC 0.000 0.071 0.000 0.071 0.071 0.214 0.143 0.071 0.214 0.071 0.071 0.000 0.000 0.000 0.000 0.000 0.000 0.000 0.000 0.000 0.000 0.000 14

LL 0.000 0.000 0.038 0.038 0.000 0.538 0.077 0.154 0.154 0.000 0.000 0.000 0.000 0.000 0.000 0.000 0.000 0.000 0.000 0.000 0.000 0.000 26

SLB 0.000 0.000 0.000 0.033 0.100 0.367 0.067 0.233 0.100 0.033 0.000 0.033 0.000 0.000 0.000 0.000 0.000 0.033 0.000 0.000 0.000 0.000 30

GL 0.000 0.000 0.000 0.000 0.115 0.385 0.038 0.231 0.154 0.000 0.000 0.038 0.000 0.000 0.038 0.000 0.000 0.000 0.000 0.000 0.000 0.000 26

DK 0.000 0.000 0.000 0.026 0.053 0.526 0.026 0.211 0.026 0.079 0.026 0.000 0.000 0.000 0.000 0.000 0.026 0.000 0.000 0.000 0.000 0.000 38

FI 0.000 0.025 0.000 0.000 0.075 0.525 0.100 0.100 0.075 0.050 0.025 0.025 0.000 0.000 0.000 0.000 0.000 0.000 0.000 0.000 0.000 0.000 40

PT 0.000 0.000 0.000 0.029 0.118 0.471 0.029 0.147 0.147 0.029 0.029 0.000 0.000 0.000 0.000 0.000 0.000 0.000 0.000 0.000 0.000 0.000 34

WK 0.000 0.000 0.000 0.000 0.029 0.618 0.118 0.059 0.147 0.000 0.000 0.029 0.000 0.000 0.000 0.000 0.000 0.000 0.000 0.000 0.000 0.000 34

CHTENG 0.000 0.050 0.000 0.100 0.100 0.400 0.100 0.200 0.050 0.000 0.000 0.000 0.000 0.000 0.000 0.000 0.000 0.000 0.000 0.000 0.000 0.000 20

Locus: 10

------------------

Pop Alleles Genes

----------------------------------------------------------------------------------------------- -----

1 2 3 4 5 6 7 8 9 10 11 12 13 14 15 16

Ad2010 0.000 0.000 0.000 0.000 0.010 0.146 0.175 0.156 0.079 0.106 0.169 0.083 0.046 0.017 0.003 0.010 302

Ad2011 0.000 0.004 0.004 0.004 0.004 0.148 0.148 0.109 0.052 0.117 0.157 0.170 0.052 0.009 0.022 0.000 230

ad2012 0.004 0.000 0.008 0.000 0.025 0.165 0.114 0.114 0.068 0.148 0.144 0.131 0.042 0.008 0.021 0.004 236

LC 0.000 0.000 0.000 0.000 0.000 0.263 0.184 0.132 0.105 0.132 0.000 0.132 0.000 0.053 0.000 0.000 38

BT 0.000 0.000 0.000 0.000 0.000 0.147 0.235 0.147 0.029 0.088 0.176 0.118 0.000 0.029 0.029 0.000 34

Q 0.000 0.000 0.000 0.000 0.000 0.115 0.231 0.038 0.115 0.231 0.077 0.115 0.038 0.038 0.000 0.000 26

CHWENG 0.000 0.000 0.000 0.000 0.000 0.300 0.000 0.100 0.100 0.200 0.000 0.000 0.200 0.100 0.000 0.000 10

CHVILL 0.000 0.000 0.000 0.000 0.000 0.000 0.333 0.000 0.000 0.333 0.167 0.000 0.167 0.000 0.000 0.000 6

CHSWE 0.000 0.000 0.000 0.000 0.000 0.156 0.094 0.156 0.125 0.188 0.094 0.062 0.031 0.000 0.094 0.000 32

CHITA 0.000 0.000 0.000 0.000 0.000 0.125 0.000 0.125 0.000 0.375 0.125 0.125 0.000 0.125 0.000 0.000 8

CHBIRL 0.000 0.000 0.000 0.000 0.000 0.214 0.036 0.179 0.107 0.143 0.179 0.107 0.000 0.036 0.000 0.000 28

BNIRL 0.000 0.000 0.000 0.000 0.000 0.125 0.083 0.083 0.208 0.292 0.042 0.042 0.083 0.000 0.042 0.000 24

SPA 0.000 0.000 0.000 0.000 0.000 0.000 0.125 0.000 0.000 0.250 0.125 0.125 0.250 0.000 0.000 0.125 8

CNIRL 0.000 0.000 0.000 0.000 0.000 0.000 0.100 0.100 0.100 0.000 0.200 0.500 0.000 0.000 0.000 0.000 10

GER 0.000 0.000 0.000 0.000 0.000 0.300 0.000 0.200 0.200 0.200 0.000 0.000 0.100 0.000 0.000 0.000 10

BU 0.000 0.000 0.000 0.000 0.000 0.094 0.125 0.125 0.094 0.125 0.188 0.156 0.062 0.031 0.000 0.000 32

BL 0.000 0.000 0.050 0.000 0.000 0.150 0.150 0.250 0.050 0.000 0.250 0.050 0.050 0.000 0.000 0.000 20

SLC 0.000 0.000 0.000 0.000 0.000 0.071 0.000 0.143 0.071 0.143 0.214 0.071 0.214 0.000 0.071 0.000 14

LL 0.000 0.000 0.000 0.000 0.000 0.269 0.115 0.077 0.000 0.038 0.077 0.154 0.192 0.000 0.077 0.000 26

SLB 0.000 0.000 0.000 0.000 0.000 0.179 0.143 0.107 0.036 0.107 0.179 0.143 0.036 0.071 0.000 0.000 28

GL 0.000 0.000 0.000 0.000 0.000 0.192 0.115 0.231 0.000 0.115 0.038 0.154 0.038 0.000 0.038 0.077 26

DK 0.000 0.000 0.000 0.000 0.000 0.132 0.289 0.132 0.079 0.132 0.105 0.079 0.053 0.000 0.000 0.000 38

FI 0.000 0.000 0.000 0.000 0.025 0.050 0.225 0.100 0.050 0.100 0.175 0.125 0.075 0.050 0.025 0.000 40

PT 0.000 0.000 0.000 0.000 0.000 0.125 0.156 0.250 0.062 0.062 0.188 0.125 0.031 0.000 0.000 0.000 32

WK 0.000 0.000 0.000 0.000 0.000 0.206 0.118 0.118 0.029 0.206 0.088 0.147 0.059 0.029 0.000 0.000 34

CHTENG 0.000 0.000 0.000 0.000 0.000 0.200 0.100 0.150 0.000 0.300 0.100 0.100 0.000 0.050 0.000 0.000 20

Locus: 11

------------------

Pop Alleles Genes

----------------------------------------------------------------------------------------------------------------------------------- -----

1 2 3 4 5 6 7 8 9 10 11 12 13 14 15 16 17 18 19 20 21 22

Ad2010 0.000 0.000 0.010 0.000 0.017 0.030 0.073 0.063 0.113 0.000 0.120 0.150 0.130 0.097 0.047 0.047 0.023 0.007 0.027 0.017 0.030 0.000 300

Ad2011 0.000 0.013 0.009 0.000 0.031 0.045 0.031 0.049 0.058 0.000 0.147 0.138 0.152 0.080 0.054 0.076 0.031 0.031 0.004 0.027 0.022 0.000 224

ad2012 0.000 0.004 0.000 0.004 0.030 0.030 0.048 0.070 0.057 0.004 0.130 0.139 0.113 0.113 0.052 0.057 0.035 0.039 0.013 0.022 0.035 0.004 230

LC 0.026 0.000 0.000 0.000 0.105 0.053 0.000 0.053 0.053 0.000 0.053 0.211 0.132 0.132 0.053 0.079 0.000 0.053 0.000 0.000 0.000 0.000 38

BT 0.000 0.000 0.000 0.000 0.000 0.029 0.000 0.118 0.088 0.000 0.088 0.176 0.088 0.118 0.088 0.088 0.059 0.029 0.029 0.000 0.000 0.000 34

Q 0.000 0.000 0.000 0.000 0.000 0.038 0.000 0.077 0.077 0.000 0.115 0.115 0.308 0.154 0.077 0.038 0.000 0.000 0.000 0.000 0.000 0.000 26

CHWENG 0.000 0.000 0.000 0.000 0.000 0.000 0.200 0.000 0.200 0.000 0.000 0.000 0.000 0.000 0.300 0.000 0.100 0.200 0.000 0.000 0.000 0.000 10

CHVILL 0.000 0.000 0.000 0.000 0.000 0.000 0.000 0.667 0.000 0.000 0.000 0.000 0.000 0.000 0.000 0.167 0.000 0.167 0.000 0.000 0.000 0.000 6

CHSWE 0.000 0.000 0.062 0.000 0.062 0.031 0.000 0.094 0.219 0.000 0.156 0.188 0.094 0.031 0.031 0.000 0.000 0.000 0.000 0.000 0.031 0.000 32

CHITA 0.000 0.000 0.000 0.000 0.000 0.000 0.000 0.000 0.000 0.000 0.250 0.250 0.250 0.000 0.000 0.000 0.000 0.000 0.000 0.000 0.250 0.000 8

CHBIRL 0.000 0.000 0.000 0.000 0.000 0.077 0.000 0.115 0.077 0.000 0.115 0.115 0.154 0.115 0.077 0.000 0.077 0.038 0.000 0.038 0.000 0.000 26

BNIRL 0.000 0.000 0.000 0.000 0.000 0.000 0.000 0.000 0.042 0.000 0.083 0.250 0.208 0.208 0.042 0.083 0.000 0.000 0.000 0.083 0.000 0.000 24

SPA 0.000 0.000 0.000 0.000 0.000 0.000 0.000 0.000 0.167 0.000 0.333 0.000 0.000 0.333 0.167 0.000 0.000 0.000 0.000 0.000 0.000 0.000 6

CNIRL 0.000 0.000 0.000 0.000 0.000 0.000 0.000 0.000 0.000 0.000 0.300 0.300 0.100 0.000 0.300 0.000 0.000 0.000 0.000 0.000 0.000 0.000 10

GER 0.000 0.000 0.000 0.000 0.000 0.200 0.000 0.000 0.200 0.000 0.000 0.000 0.100 0.100 0.300 0.100 0.000 0.000 0.000 0.000 0.000 0.000 10

BU 0.031 0.000 0.000 0.000 0.000 0.000 0.094 0.062 0.125 0.000 0.219 0.062 0.094 0.062 0.062 0.156 0.000 0.000 0.000 0.000 0.031 0.000 32

BL 0.000 0.000 0.000 0.000 0.000 0.000 0.000 0.050 0.050 0.000 0.050 0.250 0.150 0.100 0.000 0.050 0.050 0.150 0.000 0.000 0.100 0.000 20

SLC 0.000 0.000 0.083 0.000 0.000 0.083 0.000 0.083 0.167 0.000 0.083 0.083 0.167 0.167 0.000 0.083 0.000 0.000 0.000 0.000 0.000 0.000 12

LL 0.000 0.000 0.000 0.000 0.000 0.000 0.000 0.077 0.192 0.000 0.269 0.000 0.077 0.115 0.038 0.154 0.000 0.077 0.000 0.000 0.000 0.000 26

SLB 0.000 0.000 0.000 0.000 0.067 0.100 0.000 0.000 0.133 0.000 0.300 0.100 0.033 0.067 0.000 0.100 0.033 0.067 0.000 0.000 0.000 0.000 30

GL 0.000 0.000 0.038 0.000 0.038 0.000 0.038 0.038 0.115 0.000 0.154 0.231 0.077 0.077 0.000 0.038 0.077 0.000 0.077 0.000 0.000 0.000 26

DK 0.000 0.000 0.000 0.000 0.000 0.000 0.026 0.026 0.132 0.000 0.105 0.158 0.184 0.132 0.053 0.079 0.026 0.026 0.000 0.026 0.026 0.000 38

FI 0.000 0.025 0.000 0.000 0.050 0.050 0.075 0.025 0.125 0.000 0.075 0.175 0.225 0.125 0.025 0.000 0.000 0.000 0.000 0.000 0.025 0.000 40

PT 0.000 0.000 0.000 0.000 0.029 0.118 0.059 0.118 0.059 0.000 0.147 0.235 0.059 0.118 0.000 0.000 0.059 0.000 0.000 0.000 0.000 0.000 34

WK 0.000 0.000 0.000 0.000 0.029 0.147 0.029 0.059 0.088 0.000 0.059 0.235 0.029 0.088 0.000 0.118 0.000 0.000 0.029 0.000 0.088 0.000 34

CHTENG 0.000 0.000 0.000 0.000 0.000 0.050 0.000 0.100 0.200 0.000 0.050 0.150 0.000 0.200 0.100 0.000 0.000 0.050 0.000 0.100 0.000 0.000 20

Locus: 12

------------------

Pop Alleles Genes

----------------------------------------------------------------------------------------------------------------------------------------------------- -----

1 2 3 4 5 6 7 8 9 10 11 12 13 14 15 16 17 18 19 20 21 22 23 24 25

Ad2010 0.000 0.013 0.013 0.195 0.063 0.166 0.089 0.076 0.030 0.070 0.046 0.093 0.040 0.023 0.026 0.003 0.023 0.003 0.017 0.003 0.000 0.003 0.003 0.000 0.000 302

Ad2011 0.004 0.009 0.004 0.171 0.066 0.175 0.075 0.075 0.053 0.075 0.066 0.053 0.075 0.013 0.009 0.013 0.013 0.013 0.013 0.004 0.004 0.009 0.004 0.004 0.000 228

ad2012 0.000 0.004 0.000 0.200 0.062 0.221 0.108 0.067 0.054 0.050 0.021 0.050 0.033 0.037 0.017 0.017 0.004 0.025 0.008 0.004 0.000 0.004 0.008 0.000 0.004 240

LC 0.000 0.026 0.000 0.105 0.184 0.079 0.026 0.026 0.079 0.079 0.132 0.105 0.026 0.053 0.000 0.000 0.053 0.000 0.000 0.000 0.026 0.000 0.000 0.000 0.000 38

BT 0.000 0.000 0.000 0.176 0.147 0.088 0.059 0.029 0.088 0.088 0.059 0.147 0.029 0.000 0.029 0.000 0.000 0.000 0.029 0.029 0.000 0.000 0.000 0.000 0.000 34

Q 0.000 0.000 0.000 0.208 0.000 0.375 0.042 0.083 0.083 0.125 0.042 0.000 0.000 0.000 0.000 0.000 0.000 0.000 0.042 0.000 0.000 0.000 0.000 0.000 0.000 24

CHWENG 0.000 0.000 0.000 0.200 0.100 0.500 0.000 0.200 0.000 0.000 0.000 0.000 0.000 0.000 0.000 0.000 0.000 0.000 0.000 0.000 0.000 0.000 0.000 0.000 0.000 10

CHVILL 0.000 0.000 0.000 0.333 0.167 0.500 0.000 0.000 0.000 0.000 0.000 0.000 0.000 0.000 0.000 0.000 0.000 0.000 0.000 0.000 0.000 0.000 0.000 0.000 0.000 6

CHSWE 0.000 0.031 0.000 0.188 0.000 0.156 0.031 0.031 0.031 0.156 0.062 0.188 0.031 0.031 0.031 0.000 0.031 0.000 0.000 0.000 0.000 0.000 0.000 0.000 0.000 32

CHITA 0.000 0.000 0.000 0.375 0.000 0.000 0.250 0.000 0.125 0.125 0.000 0.125 0.000 0.000 0.000 0.000 0.000 0.000 0.000 0.000 0.000 0.000 0.000 0.000 0.000 8

CHBIRL 0.036 0.036 0.036 0.143 0.036 0.250 0.071 0.071 0.036 0.071 0.071 0.036 0.036 0.036 0.000 0.000 0.000 0.036 0.000 0.000 0.000 0.000 0.000 0.000 0.000 28

BNIRL 0.000 0.000 0.000 0.125 0.083 0.208 0.125 0.083 0.042 0.208 0.000 0.083 0.000 0.000 0.000 0.000 0.000 0.000 0.000 0.000 0.000 0.042 0.000 0.000 0.000 24

SPA 0.000 0.000 0.000 0.250 0.250 0.000 0.125 0.000 0.000 0.000 0.125 0.000 0.125 0.000 0.125 0.000 0.000 0.000 0.000 0.000 0.000 0.000 0.000 0.000 0.000 8

CNIRL 0.000 0.000 0.000 0.100 0.100 0.100 0.100 0.100 0.200 0.100 0.000 0.000 0.000 0.000 0.000 0.000 0.100 0.100 0.000 0.000 0.000 0.000 0.000 0.000 0.000 10

GER 0.000 0.000 0.000 0.100 0.000 0.400 0.100 0.100 0.000 0.100 0.200 0.000 0.000 0.000 0.000 0.000 0.000 0.000 0.000 0.000 0.000 0.000 0.000 0.000 0.000 10

BU 0.000 0.000 0.000 0.250 0.031 0.188 0.156 0.094 0.031 0.062 0.000 0.062 0.031 0.031 0.000 0.031 0.031 0.000 0.000 0.000 0.000 0.000 0.000 0.000 0.000 32

BL 0.000 0.000 0.000 0.150 0.100 0.200 0.200 0.050 0.050 0.050 0.050 0.050 0.050 0.000 0.000 0.000 0.000 0.000 0.000 0.000 0.000 0.000 0.000 0.050 0.000 20

SLC 0.000 0.000 0.071 0.214 0.071 0.357 0.071 0.000 0.071 0.143 0.000 0.000 0.000 0.000 0.000 0.000 0.000 0.000 0.000 0.000 0.000 0.000 0.000 0.000 0.000 14

LL 0.000 0.000 0.000 0.167 0.083 0.167 0.167 0.125 0.000 0.083 0.083 0.000 0.000 0.000 0.083 0.000 0.000 0.042 0.000 0.000 0.000 0.000 0.000 0.000 0.000 24

SLB 0.000 0.000 0.000 0.214 0.179 0.107 0.107 0.000 0.000 0.000 0.036 0.071 0.071 0.036 0.000 0.071 0.000 0.036 0.071 0.000 0.000 0.000 0.000 0.000 0.000 28

GL 0.000 0.038 0.000 0.308 0.000 0.154 0.038 0.000 0.077 0.077 0.115 0.077 0.038 0.000 0.000 0.038 0.000 0.000 0.000 0.000 0.000 0.000 0.038 0.000 0.000 26

DK 0.000 0.026 0.000 0.132 0.158 0.184 0.079 0.105 0.053 0.105 0.026 0.053 0.026 0.026 0.000 0.000 0.026 0.000 0.000 0.000 0.000 0.000 0.000 0.000 0.000 38

FI 0.000 0.000 0.000 0.175 0.050 0.300 0.050 0.050 0.025 0.050 0.100 0.100 0.000 0.025 0.025 0.025 0.000 0.000 0.025 0.000 0.000 0.000 0.000 0.000 0.000 40

PT 0.031 0.031 0.000 0.344 0.094 0.219 0.031 0.031 0.031 0.125 0.031 0.000 0.031 0.000 0.000 0.000 0.000 0.000 0.000 0.000 0.000 0.000 0.000 0.000 0.000 32

WK 0.029 0.000 0.029 0.088 0.176 0.147 0.118 0.029 0.029 0.147 0.088 0.059 0.000 0.029 0.000 0.000 0.000 0.000 0.000 0.000 0.000 0.029 0.000 0.000 0.000 34

CHTENG 0.000 0.000 0.000 0.150 0.000 0.300 0.100 0.050 0.100 0.000 0.050 0.100 0.100 0.000 0.000 0.000 0.000 0.000 0.050 0.000 0.000 0.000 0.000 0.000 0.000 20

Locus: 13

------------------

Pop Alleles Genes

----------------------------------------------------------------------------------------------------- -----

1 2 3 4 5 6 7 8 9 10 11 12 13 14 15 16 17

Ad2010 0.003 0.007 0.000 0.086 0.017 0.169 0.020 0.285 0.050 0.169 0.063 0.050 0.063 0.010 0.007 0.000 0.003 302

Ad2011 0.009 0.013 0.013 0.096 0.018 0.110 0.066 0.237 0.145 0.114 0.123 0.018 0.031 0.004 0.004 0.000 0.000 228

ad2012 0.004 0.009 0.004 0.075 0.031 0.154 0.022 0.254 0.057 0.154 0.105 0.044 0.044 0.004 0.026 0.004 0.009 228

LC 0.000 0.000 0.000 0.118 0.029 0.176 0.029 0.294 0.029 0.029 0.088 0.118 0.088 0.000 0.000 0.000 0.000 34

BT 0.000 0.000 0.000 0.147 0.000 0.147 0.000 0.206 0.176 0.176 0.059 0.029 0.000 0.000 0.059 0.000 0.000 34

Q 0.000 0.000 0.000 0.208 0.042 0.250 0.000 0.125 0.042 0.125 0.042 0.000 0.125 0.000 0.000 0.042 0.000 24

CHWENG 0.000 0.000 0.000 0.000 0.000 0.200 0.000 0.300 0.000 0.400 0.000 0.000 0.100 0.000 0.000 0.000 0.000 10

CHVILL 0.000 0.000 0.000 0.000 0.000 0.500 0.000 0.000 0.000 0.000 0.250 0.000 0.250 0.000 0.000 0.000 0.000 4

CHSWE 0.000 0.000 0.000 0.062 0.000 0.094 0.031 0.438 0.094 0.031 0.188 0.031 0.031 0.000 0.000 0.000 0.000 32

CHITA 0.000 0.000 0.000 0.125 0.000 0.125 0.000 0.125 0.000 0.125 0.500 0.000 0.000 0.000 0.000 0.000 0.000 8

CHBIRL 0.000 0.036 0.000 0.143 0.000 0.143 0.000 0.214 0.107 0.179 0.107 0.000 0.071 0.000 0.000 0.000 0.000 28

BNIRL 0.000 0.000 0.000 0.000 0.042 0.167 0.042 0.167 0.083 0.375 0.042 0.042 0.042 0.000 0.000 0.000 0.000 24

SPA 0.000 0.000 0.000 0.000 0.000 0.250 0.000 0.125 0.000 0.250 0.250 0.000 0.125 0.000 0.000 0.000 0.000 8

CNIRL 0.000 0.000 0.000 0.000 0.000 0.200 0.100 0.400 0.000 0.100 0.000 0.100 0.100 0.000 0.000 0.000 0.000 10

GER 0.000 0.000 0.000 0.200 0.000 0.100 0.000 0.200 0.100 0.200 0.100 0.100 0.000 0.000 0.000 0.000 0.000 10

BU 0.000 0.031 0.000 0.094 0.031 0.094 0.031 0.250 0.094 0.188 0.031 0.031 0.094 0.000 0.000 0.000 0.031 32

BL 0.000 0.000 0.000 0.056 0.056 0.222 0.000 0.389 0.000 0.167 0.111 0.000 0.000 0.000 0.000 0.000 0.000 18

SLC 0.000 0.071 0.000 0.143 0.000 0.071 0.000 0.500 0.000 0.071 0.143 0.000 0.000 0.000 0.000 0.000 0.000 14

LL 0.000 0.000 0.000 0.038 0.000 0.231 0.000 0.231 0.154 0.115 0.154 0.000 0.000 0.038 0.000 0.038 0.000 26

SLB 0.000 0.000 0.000 0.045 0.136 0.045 0.045 0.227 0.091 0.091 0.182 0.045 0.045 0.000 0.045 0.000 0.000 22

GL 0.000 0.042 0.000 0.083 0.000 0.083 0.042 0.333 0.125 0.125 0.083 0.000 0.042 0.000 0.042 0.000 0.000 24

DK 0.000 0.026 0.000 0.053 0.000 0.237 0.079 0.289 0.105 0.053 0.026 0.026 0.105 0.000 0.000 0.000 0.000 38

FI 0.000 0.000 0.029 0.118 0.000 0.176 0.000 0.088 0.206 0.147 0.088 0.000 0.029 0.088 0.000 0.029 0.000 34

PT 0.000 0.036 0.036 0.107 0.071 0.143 0.071 0.250 0.107 0.071 0.036 0.000 0.036 0.000 0.036 0.000 0.000 28

WK 0.000 0.077 0.000 0.115 0.000 0.115 0.115 0.308 0.231 0.000 0.038 0.000 0.000 0.000 0.000 0.000 0.000 26

CHTENG 0.000 0.000 0.000 0.100 0.000 0.150 0.000 0.200 0.050 0.250 0.100 0.000 0.100 0.000 0.000 0.000 0.050 20

Locus: 14

------------------

Pop Alleles Genes

----------------------------------------------- -----

1 2 3 4 5 6 7 8

Ad2010 0.000 0.012 0.086 0.254 0.586 0.025 0.025 0.012 244

Ad2011 0.000 0.005 0.135 0.266 0.536 0.041 0.005 0.014 222

ad2012 0.004 0.013 0.049 0.326 0.580 0.022 0.000 0.004 224

LC 0.000 0.000 0.000 0.250 0.688 0.031 0.031 0.000 32

BT 0.000 0.000 0.088 0.324 0.529 0.000 0.059 0.000 34

Q 0.000 0.000 0.000 0.409 0.545 0.045 0.000 0.000 22

CHWENG 0.000 0.000 0.000 0.600 0.400 0.000 0.000 0.000 10

CHVILL 0.000 0.000 0.000 0.250 0.750 0.000 0.000 0.000 4

CHSWE 0.031 0.031 0.062 0.312 0.531 0.031 0.000 0.000 32

CHITA 0.000 0.000 0.000 0.250 0.625 0.125 0.000 0.000 8

CHBIRL 0.000 0.000 0.000 0.346 0.500 0.038 0.038 0.077 26

BNIRL 0.000 0.000 0.182 0.045 0.682 0.045 0.000 0.045 22

SPA 0.000 0.000 0.000 0.375 0.625 0.000 0.000 0.000 8

CNIRL 0.000 0.000 0.000 0.700 0.100 0.200 0.000 0.000 10

GER 0.000 0.000 0.000 0.000 0.800 0.200 0.000 0.000 10

BU 0.000 0.000 0.031 0.312 0.625 0.031 0.000 0.000 32

BL 0.000 0.000 0.000 0.056 0.944 0.000 0.000 0.000 18

SLC 0.000 0.071 0.000 0.500 0.429 0.000 0.000 0.000 14

LL 0.000 0.000 0.000 0.500 0.500 0.000 0.000 0.000 22

SLB 0.000 0.045 0.000 0.500 0.455 0.000 0.000 0.000 22

GL 0.000 0.045 0.045 0.364 0.455 0.091 0.000 0.000 22

DK 0.000 0.000 0.000 0.553 0.395 0.053 0.000 0.000 38

FI 0.000 0.000 0.062 0.406 0.531 0.000 0.000 0.000 32

PT 0.000 0.067 0.100 0.233 0.533 0.000 0.067 0.000 30

WK 0.000 0.000 0.077 0.269 0.615 0.000 0.038 0.000 26

CHTENG 0.050 0.000 0.000 0.300 0.600 0.050 0.000 0.000 20

Locus: 15

------------------

Pop Alleles Genes

----------------------------------------------------------------------------------------------------- -----

1 2 3 4 5 6 7 8 9 10 11 12 13 14 15 16 17

Ad2010 0.033 0.010 0.000 0.020 0.010 0.003 0.007 0.003 0.007 0.007 0.103 0.318 0.364 0.050 0.043 0.013 0.010 302

Ad2011 0.101 0.022 0.004 0.018 0.018 0.000 0.009 0.009 0.004 0.026 0.048 0.268 0.320 0.088 0.026 0.026 0.013 228

ad2012 0.080 0.027 0.009 0.009 0.009 0.000 0.000 0.018 0.009 0.013 0.115 0.274 0.305 0.053 0.044 0.035 0.000 226

LC 0.059 0.029 0.029 0.000 0.029 0.000 0.000 0.000 0.029 0.000 0.059 0.412 0.147 0.059 0.088 0.059 0.000 34

BT 0.029 0.000 0.029 0.000 0.029 0.000 0.000 0.000 0.000 0.000 0.059 0.382 0.382 0.000 0.059 0.029 0.000 34

Q 0.042 0.000 0.000 0.083 0.000 0.000 0.000 0.000 0.000 0.000 0.042 0.375 0.208 0.167 0.042 0.042 0.000 24

CHWENG 0.100 0.000 0.000 0.000 0.000 0.000 0.000 0.000 0.000 0.000 0.300 0.200 0.100 0.100 0.000 0.200 0.000 10

CHVILL 0.000 0.000 0.000 0.250 0.000 0.000 0.000 0.000 0.000 0.000 0.000 0.750 0.000 0.000 0.000 0.000 0.000 4

CHSWE 0.094 0.000 0.000 0.062 0.000 0.000 0.000 0.000 0.000 0.000 0.094 0.250 0.281 0.094 0.125 0.000 0.000 32

CHITA 0.000 0.000 0.000 0.000 0.000 0.000 0.000 0.000 0.000 0.000 0.250 0.500 0.250 0.000 0.000 0.000 0.000 8

CHBIRL 0.038 0.115 0.038 0.000 0.000 0.000 0.038 0.000 0.000 0.000 0.115 0.192 0.308 0.077 0.077 0.000 0.000 26

BNIRL 0.000 0.000 0.000 0.000 0.000 0.000 0.000 0.000 0.000 0.000 0.091 0.500 0.273 0.045 0.091 0.000 0.000 22

SPA 0.000 0.000 0.000 0.000 0.000 0.000 0.000 0.000 0.000 0.000 0.125 0.375 0.500 0.000 0.000 0.000 0.000 8

CNIRL 0.100 0.100 0.000 0.000 0.000 0.000 0.000 0.000 0.000 0.100 0.100 0.100 0.300 0.200 0.000 0.000 0.000 10

GER 0.100 0.200 0.000 0.000 0.100 0.000 0.000 0.000 0.000 0.000 0.100 0.100 0.300 0.000 0.100 0.000 0.000 10

BU 0.062 0.000 0.000 0.031 0.000 0.000 0.031 0.000 0.000 0.031 0.094 0.344 0.219 0.031 0.062 0.062 0.031 32

BL 0.200 0.000 0.000 0.000 0.100 0.000 0.000 0.000 0.000 0.000 0.000 0.300 0.200 0.100 0.050 0.050 0.000 20

SLC 0.214 0.071 0.000 0.000 0.000 0.000 0.000 0.000 0.000 0.000 0.000 0.357 0.071 0.214 0.071 0.000 0.000 14

LL 0.077 0.000 0.000 0.000 0.000 0.000 0.000 0.038 0.000 0.000 0.000 0.500 0.385 0.000 0.000 0.000 0.000 26

SLB 0.182 0.000 0.000 0.045 0.000 0.000 0.000 0.000 0.000 0.045 0.182 0.182 0.227 0.045 0.091 0.000 0.000 22

GL 0.042 0.042 0.000 0.042 0.042 0.000 0.000 0.000 0.000 0.042 0.083 0.292 0.125 0.000 0.250 0.000 0.042 24

DK 0.053 0.053 0.026 0.079 0.000 0.000 0.000 0.000 0.000 0.026 0.053 0.316 0.316 0.053 0.026 0.000 0.000 38

FI 0.059 0.000 0.000 0.000 0.000 0.000 0.000 0.000 0.000 0.000 0.029 0.441 0.324 0.088 0.059 0.000 0.000 34

PT 0.133 0.067 0.000 0.000 0.000 0.000 0.000 0.000 0.000 0.033 0.067 0.233 0.367 0.100 0.000 0.000 0.000 30

WK 0.038 0.000 0.000 0.038 0.000 0.000 0.038 0.000 0.000 0.000 0.077 0.423 0.192 0.154 0.038 0.000 0.000 26

CHTENG 0.100 0.000 0.000 0.050 0.000 0.000 0.000 0.000 0.000 0.050 0.100 0.150 0.400 0.150 0.000 0.000 0.000 20

Locus: 16

------------------

Pop Alleles Genes

----------------------------------------- -----

1 2 3 4 5 6 7

Ad2010 0.003 0.000 0.010 0.921 0.043 0.023 0.000 302

Ad2011 0.000 0.000 0.004 0.930 0.066 0.000 0.000 228

ad2012 0.000 0.004 0.039 0.890 0.053 0.013 0.000 228

LC 0.000 0.000 0.000 0.906 0.062 0.031 0.000 32

BT 0.000 0.000 0.000 0.971 0.029 0.000 0.000 34

Q 0.000 0.000 0.042 0.833 0.125 0.000 0.000 24

CHWENG 0.000 0.000 0.000 0.700 0.300 0.000 0.000 10

CHVILL 0.000 0.000 0.000 0.750 0.000 0.250 0.000 4

CHSWE 0.000 0.000 0.000 0.969 0.000 0.031 0.000 32

CHITA 0.000 0.000 0.000 1.000 0.000 0.000 0.000 8

CHBIRL 0.000 0.000 0.000 0.893 0.107 0.000 0.000 28

BNIRL 0.000 0.000 0.000 1.000 0.000 0.000 0.000 22

SPA 0.000 0.000 0.000 1.000 0.000 0.000 0.000 8

CNIRL 0.000 0.000 0.000 1.000 0.000 0.000 0.000 10

GER 0.000 0.000 0.000 1.000 0.000 0.000 0.000 10

BU 0.000 0.031 0.000 0.938 0.031 0.000 0.000 32

BL 0.000 0.000 0.056 0.778 0.167 0.000 0.000 18

SLC 0.000 0.000 0.071 0.929 0.000 0.000 0.000 14

LL 0.000 0.000 0.000 0.917 0.083 0.000 0.000 24

SLB 0.000 0.000 0.000 0.955 0.000 0.045 0.000 22

GL 0.000 0.000 0.000 0.958 0.042 0.000 0.000 24

DK 0.000 0.000 0.026 0.816 0.132 0.000 0.026 38

FI 0.000 0.000 0.000 0.938 0.031 0.031 0.000 32

PT 0.033 0.000 0.033 0.867 0.067 0.000 0.000 30

WK 0.000 0.000 0.000 0.923 0.077 0.000 0.000 26

CHTENG 0.000 0.000 0.000 0.800 0.150 0.000 0.050 20

Locus: 17

------------------

Pop Alleles Genes

----------------------------------------------------------------------------------- -----

1 2 3 4 5 6 7 8 9 10 11 12 13 14

Ad2010 0.000 0.019 0.087 0.091 0.098 0.053 0.053 0.080 0.038 0.106 0.038 0.133 0.163 0.042 264

Ad2011 0.000 0.036 0.054 0.063 0.077 0.086 0.068 0.063 0.050 0.086 0.032 0.194 0.180 0.014 222

ad2012 0.009 0.041 0.068 0.118 0.100 0.091 0.118 0.073 0.073 0.077 0.014 0.077 0.105 0.036 220

LC 0.000 0.267 0.067 0.100 0.167 0.033 0.067 0.067 0.067 0.067 0.000 0.000 0.000 0.100 30

BT 0.000 0.000 0.031 0.156 0.281 0.031 0.156 0.094 0.062 0.125 0.000 0.000 0.062 0.000 32

Q 0.000 0.091 0.091 0.000 0.182 0.000 0.045 0.091 0.136 0.227 0.000 0.000 0.091 0.045 22

CHWENG 0.000 0.000 0.100 0.100 0.200 0.000 0.100 0.200 0.200 0.000 0.100 0.000 0.000 0.000 10

CHVILL 0.000 0.000 0.000 0.250 0.000 0.000 0.250 0.250 0.000 0.000 0.250 0.000 0.000 0.000 4

CHSWE 0.000 0.031 0.062 0.000 0.000 0.062 0.031 0.031 0.031 0.125 0.031 0.375 0.219 0.000 32

CHITA 0.000 0.125 0.000 0.000 0.000 0.000 0.000 0.125 0.125 0.000 0.000 0.250 0.375 0.000 8

CHBIRL 0.000 0.000 0.038 0.077 0.077 0.077 0.231 0.000 0.077 0.115 0.000 0.115 0.192 0.000 26

BNIRL 0.000 0.045 0.045 0.045 0.045 0.045 0.091 0.136 0.045 0.045 0.045 0.091 0.318 0.000 22

SPA 0.000 0.000 0.000 0.125 0.125 0.250 0.000 0.000 0.000 0.000 0.125 0.125 0.250 0.000 8

CNIRL 0.000 0.000 0.100 0.000 0.000 0.000 0.200 0.100 0.000 0.000 0.000 0.600 0.000 0.000 10

GER 0.000 0.100 0.000 0.100 0.000 0.000 0.000 0.000 0.000 0.200 0.000 0.000 0.600 0.000 10

BU 0.000 0.033 0.167 0.033 0.100 0.200 0.100 0.067 0.033 0.067 0.000 0.167 0.033 0.000 30

BL 0.000 0.222 0.000 0.111 0.111 0.000 0.056 0.056 0.111 0.222 0.056 0.056 0.000 0.000 18

SLC 0.000 0.000 0.143 0.000 0.286 0.071 0.000 0.143 0.000 0.000 0.143 0.000 0.071 0.143 14

LL 0.000 0.136 0.273 0.000 0.000 0.091 0.091 0.000 0.045 0.045 0.091 0.000 0.091 0.136 22

SLB 0.000 0.318 0.091 0.045 0.000 0.000 0.227 0.045 0.091 0.136 0.000 0.000 0.045 0.000 22

GL 0.000 0.000 0.136 0.045 0.000 0.182 0.045 0.091 0.045 0.273 0.091 0.000 0.045 0.045 22

DK 0.000 0.094 0.062 0.062 0.094 0.000 0.094 0.219 0.000 0.125 0.094 0.031 0.094 0.031 32

FI 0.000 0.176 0.088 0.088 0.059 0.088 0.176 0.029 0.000 0.235 0.029 0.000 0.029 0.000 34

PT 0.000 0.000 0.154 0.115 0.077 0.115 0.038 0.038 0.154 0.115 0.000 0.077 0.077 0.038 26

WK 0.000 0.045 0.182 0.000 0.045 0.182 0.045 0.091 0.182 0.091 0.000 0.091 0.045 0.000 22

CHTENG 0.000 0.050 0.050 0.050 0.050 0.050 0.000 0.100 0.150 0.000 0.000 0.250 0.250 0.000 20

Locus: 18

------------------

Pop Alleles Genes

----------------------------------------- -----

1 2 3 4 5 6 7

Ad2010 0.000 0.000 0.000 0.003 0.020 0.977 0.000 302

Ad2011 0.009 0.000 0.000 0.009 0.039 0.934 0.009 228

ad2012 0.000 0.004 0.000 0.004 0.054 0.933 0.004 224

LC 0.000 0.000 0.000 0.000 0.000 1.000 0.000 32

BT 0.000 0.000 0.000 0.000 0.088 0.912 0.000 34

Q 0.042 0.000 0.000 0.000 0.000 0.958 0.000 24

CHWENG 0.000 0.000 0.000 0.000 0.100 0.900 0.000 10

CHVILL 0.000 0.000 0.000 0.000 0.000 1.000 0.000 4

CHSWE 0.000 0.000 0.000 0.000 0.000 1.000 0.000 32

CHITA 0.000 0.000 0.000 0.000 0.000 1.000 0.000 8

CHBIRL 0.000 0.000 0.000 0.000 0.000 1.000 0.000 26

BNIRL 0.000 0.000 0.000 0.000 0.000 1.000 0.000 22

SPA 0.000 0.125 0.000 0.000 0.000 0.875 0.000 8

CNIRL 0.000 0.000 0.000 0.000 0.000 1.000 0.000 10

GER 0.000 0.000 0.000 0.000 0.000 1.000 0.000 10

BU 0.000 0.000 0.000 0.000 0.000 1.000 0.000 32

BL 0.000 0.000 0.000 0.000 0.000 1.000 0.000 18

SLC 0.000 0.000 0.000 0.000 0.071 0.929 0.000 14

LL 0.000 0.000 0.000 0.000 0.000 1.000 0.000 22

SLB 0.000 0.000 0.000 0.000 0.000 0.955 0.045 22

GL 0.000 0.000 0.000 0.000 0.042 0.958 0.000 24

DK 0.000 0.000 0.028 0.000 0.056 0.917 0.000 36

FI 0.000 0.000 0.000 0.000 0.094 0.906 0.000 32

PT 0.000 0.000 0.000 0.000 0.167 0.833 0.000 30

WK 0.000 0.000 0.000 0.000 0.077 0.885 0.038 26

CHTENG 0.000 0.000 0.000 0.000 0.000 1.000 0.000 20

Locus: 19

------------------

Pop Alleles Genes

----------------------------------------------------------------------------------------------------------------- -----

1 2 3 4 5 6 7 8 9 10 11 12 13 14 15 16 17 18 19

Ad2010 0.000 0.007 0.013 0.109 0.146 0.341 0.099 0.103 0.026 0.079 0.026 0.007 0.017 0.010 0.003 0.003 0.000 0.007 0.003 302

Ad2011 0.000 0.013 0.018 0.088 0.164 0.438 0.075 0.062 0.040 0.053 0.018 0.000 0.009 0.004 0.009 0.000 0.004 0.000 0.004 226

ad2012 0.000 0.025 0.042 0.125 0.163 0.371 0.083 0.062 0.025 0.046 0.017 0.008 0.004 0.008 0.004 0.008 0.004 0.004 0.000 240

LC 0.000 0.000 0.132 0.105 0.053 0.474 0.132 0.026 0.026 0.000 0.000 0.000 0.026 0.000 0.000 0.026 0.000 0.000 0.000 38

BT 0.000 0.029 0.147 0.176 0.088 0.206 0.118 0.059 0.000 0.147 0.000 0.000 0.000 0.000 0.000 0.000 0.000 0.029 0.000 34

Q 0.000 0.077 0.308 0.000 0.077 0.346 0.154 0.000 0.000 0.038 0.000 0.000 0.000 0.000 0.000 0.000 0.000 0.000 0.000 26

CHWENG 0.000 0.000 0.000 0.300 0.300 0.100 0.000 0.200 0.000 0.000 0.000 0.100 0.000 0.000 0.000 0.000 0.000 0.000 0.000 10

CHVILL 0.000 0.000 0.000 0.167 0.333 0.167 0.167 0.000 0.000 0.000 0.000 0.000 0.167 0.000 0.000 0.000 0.000 0.000 0.000 6

CHSWE 0.000 0.031 0.000 0.031 0.156 0.406 0.125 0.094 0.000 0.125 0.000 0.031 0.000 0.000 0.000 0.000 0.000 0.000 0.000 32

CHITA 0.000 0.000 0.000 0.000 0.250 0.000 0.375 0.000 0.000 0.000 0.000 0.000 0.250 0.000 0.000 0.125 0.000 0.000 0.000 8

CHBIRL 0.000 0.000 0.000 0.071 0.357 0.357 0.143 0.000 0.036 0.000 0.036 0.000 0.000 0.000 0.000 0.000 0.000 0.000 0.000 28

BNIRL 0.000 0.000 0.000 0.227 0.364 0.318 0.000 0.091 0.000 0.000 0.000 0.000 0.000 0.000 0.000 0.000 0.000 0.000 0.000 22

SPA 0.000 0.000 0.000 0.250 0.250 0.125 0.125 0.000 0.000 0.125 0.000 0.125 0.000 0.000 0.000 0.000 0.000 0.000 0.000 8

CNIRL 0.000 0.000 0.000 0.000 0.000 0.600 0.300 0.000 0.000 0.100 0.000 0.000 0.000 0.000 0.000 0.000 0.000 0.000 0.000 10

GER 0.000 0.000 0.100 0.100 0.100 0.200 0.300 0.100 0.100 0.000 0.000 0.000 0.000 0.000 0.000 0.000 0.000 0.000 0.000 10

BU 0.000 0.000 0.062 0.062 0.031 0.375 0.125 0.094 0.000 0.188 0.031 0.000 0.000 0.031 0.000 0.000 0.000 0.000 0.000 32

BL 0.000 0.000 0.000 0.000 0.200 0.450 0.150 0.050 0.050 0.000 0.100 0.000 0.000 0.000 0.000 0.000 0.000 0.000 0.000 20

SLC 0.000 0.000 0.000 0.000 0.143 0.214 0.071 0.429 0.000 0.071 0.000 0.000 0.000 0.071 0.000 0.000 0.000 0.000 0.000 14

LL 0.077 0.000 0.192 0.038 0.038 0.385 0.192 0.000 0.000 0.038 0.000 0.000 0.038 0.000 0.000 0.000 0.000 0.000 0.000 26

SLB 0.000 0.000 0.107 0.071 0.143 0.357 0.143 0.071 0.000 0.036 0.036 0.036 0.000 0.000 0.000 0.000 0.000 0.000 0.000 28

GL 0.000 0.000 0.000 0.091 0.136 0.455 0.182 0.045 0.045 0.000 0.000 0.000 0.000 0.000 0.045 0.000 0.000 0.000 0.000 22

DK 0.000 0.000 0.000 0.194 0.139 0.250 0.139 0.167 0.000 0.028 0.056 0.028 0.000 0.000 0.000 0.000 0.000 0.000 0.000 36

FI 0.000 0.000 0.053 0.053 0.105 0.421 0.184 0.105 0.000 0.000 0.053 0.000 0.000 0.000 0.000 0.000 0.000 0.000 0.026 38

PT 0.000 0.000 0.000 0.071 0.286 0.250 0.214 0.071 0.000 0.000 0.071 0.000 0.000 0.000 0.000 0.000 0.036 0.000 0.000 28

WK 0.000 0.000 0.000 0.100 0.233 0.400 0.100 0.033 0.000 0.033 0.000 0.000 0.100 0.000 0.000 0.000 0.000 0.000 0.000 30

CHTENG 0.000 0.000 0.000 0.000 0.250 0.400 0.050 0.100 0.000 0.150 0.050 0.000 0.000 0.000 0.000 0.000 0.000 0.000 0.000 20

Locus: 20

------------------

Pop Alleles Genes

----------------------------------------------------------------- -----

1 2 3 4 5 6 7 8 9 10 11

Ad2010 0.050 0.047 0.076 0.299 0.133 0.162 0.112 0.054 0.054 0.014 0.000 278

Ad2011 0.081 0.043 0.043 0.276 0.105 0.152 0.186 0.067 0.033 0.014 0.000 210

ad2012 0.065 0.039 0.134 0.263 0.147 0.116 0.155 0.030 0.017 0.034 0.000 232

LC 0.000 0.132 0.053 0.395 0.026 0.105 0.132 0.105 0.053 0.000 0.000 38

BT 0.029 0.059 0.294 0.265 0.118 0.059 0.176 0.000 0.000 0.000 0.000 34

Q 0.000 0.125 0.083 0.375 0.083 0.083 0.167 0.042 0.000 0.042 0.000 24

CHWENG 0.000 0.000 0.000 0.600 0.000 0.000 0.100 0.200 0.000 0.100 0.000 10

CHVILL 0.000 0.000 0.167 0.167 0.167 0.000 0.333 0.167 0.000 0.000 0.000 6

CHSWE 0.000 0.125 0.125 0.219 0.062 0.000 0.250 0.062 0.062 0.094 0.000 32

CHITA 0.000 0.000 0.250 0.250 0.250 0.000 0.250 0.000 0.000 0.000 0.000 8

CHBIRL 0.000 0.143 0.000 0.429 0.321 0.000 0.036 0.071 0.000 0.000 0.000 28

BNIRL 0.000 0.125 0.083 0.125 0.167 0.000 0.333 0.083 0.000 0.083 0.000 24

SPA 0.000 0.000 0.000 0.000 0.375 0.000 0.125 0.250 0.000 0.250 0.000 8

CNIRL 0.000 0.000 0.000 0.400 0.100 0.100 0.100 0.100 0.200 0.000 0.000 10

GER 0.000 0.100 0.000 0.500 0.000 0.000 0.000 0.000 0.200 0.200 0.000 10

BU 0.031 0.094 0.125 0.312 0.125 0.094 0.125 0.031 0.031 0.031 0.000 32

BL 0.050 0.050 0.100 0.250 0.150 0.150 0.100 0.050 0.050 0.050 0.000 20

SLC 0.100 0.000 0.400 0.100 0.000 0.000 0.200 0.200 0.000 0.000 0.000 10

LL 0.038 0.000 0.154 0.192 0.231 0.115 0.115 0.115 0.000 0.038 0.000 26

SLB 0.071 0.071 0.143 0.321 0.107 0.143 0.107 0.036 0.000 0.000 0.000 28

GL 0.045 0.182 0.182 0.182 0.136 0.136 0.000 0.091 0.045 0.000 0.000 22

DK 0.000 0.083 0.111 0.222 0.056 0.278 0.139 0.056 0.056 0.000 0.000 36

FI 0.079 0.026 0.132 0.263 0.053 0.184 0.158 0.053 0.000 0.026 0.026 38

PT 0.000 0.100 0.100 0.233 0.067 0.167 0.133 0.067 0.067 0.067 0.000 30

WK 0.067 0.000 0.100 0.333 0.067 0.033 0.233 0.100 0.033 0.033 0.000 30

CHTENG 0.050 0.000 0.150 0.350 0.150 0.000 0.100 0.100 0.100 0.000 0.000 20

Locus: 21

------------------

Pop Alleles Genes

----------------------------------------------------------------- -----

1 2 3 4 5 6 7 8 9 10 11

Ad2010 0.003 0.000 0.014 0.010 0.116 0.320 0.235 0.136 0.109 0.041 0.017 294

Ad2011 0.014 0.018 0.032 0.045 0.090 0.261 0.167 0.135 0.113 0.081 0.045 222

ad2012 0.009 0.013 0.004 0.009 0.091 0.289 0.181 0.181 0.112 0.039 0.073 232

LC 0.000 0.000 0.000 0.028 0.167 0.167 0.306 0.167 0.083 0.083 0.000 36

BT 0.031 0.062 0.000 0.031 0.156 0.219 0.188 0.094 0.031 0.125 0.062 32

Q 0.000 0.077 0.000 0.000 0.154 0.269 0.231 0.000 0.192 0.000 0.077 26

CHWENG 0.000 0.000 0.000 0.000 0.100 0.100 0.300 0.200 0.000 0.200 0.100 10

CHVILL 0.000 0.000 0.000 0.000 0.000 0.250 0.000 0.250 0.000 0.500 0.000 4

CHSWE 0.000 0.031 0.000 0.000 0.062 0.469 0.188 0.062 0.031 0.094 0.062 32

CHITA 0.000 0.000 0.000 0.250 0.125 0.250 0.000 0.250 0.125 0.000 0.000 8

CHBIRL 0.000 0.071 0.000 0.000 0.107 0.286 0.286 0.071 0.179 0.000 0.000 28

BNIRL 0.000 0.000 0.000 0.000 0.083 0.292 0.208 0.083 0.292 0.042 0.000 24

SPA 0.000 0.000 0.000 0.000 0.000 0.000 0.167 0.333 0.167 0.000 0.333 6

CNIRL 0.100 0.000 0.000 0.000 0.200 0.400 0.000 0.000 0.300 0.000 0.000 10

GER 0.000 0.000 0.000 0.000 0.300 0.000 0.200 0.100 0.200 0.200 0.000 10

BU 0.000 0.000 0.000 0.067 0.100 0.200 0.233 0.100 0.167 0.067 0.067 30

BL 0.050 0.000 0.000 0.000 0.150 0.150 0.300 0.300 0.000 0.000 0.050 20

SLC 0.000 0.000 0.071 0.000 0.071 0.286 0.143 0.214 0.071 0.071 0.071 14

LL 0.000 0.000 0.000 0.000 0.077 0.308 0.269 0.077 0.115 0.115 0.038 26

SLB 0.000 0.000 0.000 0.000 0.067 0.400 0.267 0.067 0.067 0.033 0.100 30

GL 0.091 0.000 0.000 0.000 0.000 0.227 0.273 0.227 0.000 0.182 0.000 22

DK 0.000 0.000 0.000 0.000 0.184 0.289 0.237 0.079 0.105 0.026 0.079 38

FI 0.000 0.000 0.026 0.026 0.132 0.316 0.211 0.053 0.132 0.000 0.105 38

PT 0.000 0.000 0.000 0.000 0.167 0.167 0.200 0.167 0.000 0.200 0.100 30

WK 0.000 0.000 0.000 0.000 0.133 0.300 0.267 0.100 0.100 0.000 0.100 30

CHTENG 0.000 0.000 0.000 0.000 0.100 0.450 0.300 0.000 0.050 0.100 0.000 20

Locus: 22

------------------

Pop Alleles Genes

----------------------------------------------------------------------------------------------------------------------- -----

1 2 3 4 5 6 7 8 9 10 11 12 13 14 15 16 17 18 19 20

Ad2010 0.003 0.000 0.043 0.000 0.047 0.300 0.023 0.047 0.020 0.010 0.023 0.027 0.063 0.033 0.063 0.090 0.063 0.077 0.033 0.033 300

Ad2011 0.000 0.000 0.053 0.009 0.031 0.288 0.027 0.027 0.027 0.018 0.013 0.066 0.035 0.049 0.066 0.066 0.088 0.102 0.022 0.013 226

ad2012 0.000 0.000 0.004 0.000 0.035 0.272 0.048 0.026 0.044 0.004 0.009 0.031 0.066 0.044 0.096 0.110 0.101 0.061 0.039 0.009 228

LC 0.000 0.000 0.029 0.000 0.029 0.353 0.000 0.000 0.088 0.000 0.029 0.000 0.118 0.000 0.088 0.088 0.088 0.029 0.059 0.000 34

BT 0.000 0.000 0.000 0.000 0.000 0.462 0.038 0.000 0.038 0.038 0.000 0.038 0.038 0.038 0.077 0.077 0.000 0.115 0.038 0.000 26

Q 0.000 0.000 0.000 0.000 0.050 0.100 0.050 0.100 0.050 0.000 0.000 0.000 0.050 0.150 0.200 0.050 0.100 0.100 0.000 0.000 20

CHWENG 0.000 0.000 0.000 0.000 0.000 0.300 0.000 0.000 0.300 0.000 0.000 0.100 0.000 0.000 0.100 0.100 0.000 0.000 0.000 0.100 10

CHVILL 0.000 0.000 0.000 0.000 0.167 0.000 0.000 0.167 0.000 0.000 0.000 0.000 0.000 0.000 0.500 0.000 0.000 0.167 0.000 0.000 6

CHSWE 0.000 0.031 0.000 0.000 0.125 0.094 0.000 0.062 0.000 0.000 0.062 0.094 0.094 0.094 0.000 0.188 0.156 0.000 0.000 0.000 32

CHITA 0.000 0.000 0.000 0.000 0.000 0.125 0.000 0.250 0.000 0.000 0.000 0.125 0.125 0.000 0.250 0.000 0.125 0.000 0.000 0.000 8

CHBIRL 0.000 0.000 0.000 0.000 0.000 0.321 0.036 0.071 0.000 0.143 0.000 0.036 0.000 0.000 0.179 0.143 0.036 0.000 0.000 0.036 28

BNIRL 0.000 0.000 0.042 0.000 0.042 0.333 0.042 0.000 0.125 0.000 0.000 0.000 0.083 0.042 0.125 0.083 0.000 0.083 0.000 0.000 24

SPA 0.000 0.000 0.000 0.000 0.000 0.125 0.000 0.000 0.000 0.000 0.000 0.000 0.000 0.000 0.125 0.375 0.375 0.000 0.000 0.000 8

CNIRL 0.000 0.000 0.000 0.000 0.000 0.300 0.000 0.000 0.000 0.100 0.000 0.000 0.000 0.100 0.200 0.100 0.000 0.100 0.000 0.100 10

GER 0.000 0.000 0.000 0.000 0.000 0.300 0.000 0.000 0.000 0.000 0.000 0.000 0.000 0.000 0.000 0.300 0.300 0.100 0.000 0.000 10

BU 0.000 0.000 0.000 0.000 0.000 0.200 0.100 0.067 0.000 0.000 0.033 0.067 0.133 0.067 0.033 0.033 0.133 0.133 0.000 0.000 30

BL 0.000 0.000 0.000 0.000 0.000 0.250 0.000 0.062 0.000 0.000 0.062 0.125 0.125 0.125 0.062 0.062 0.062 0.000 0.062 0.000 16

SLC 0.000 0.000 0.000 0.000 0.000 0.143 0.000 0.000 0.071 0.000 0.071 0.071 0.071 0.000 0.000 0.143 0.286 0.143 0.000 0.000 14

LL 0.000 0.000 0.000 0.000 0.000 0.292 0.000 0.042 0.042 0.000 0.000 0.083 0.042 0.000 0.083 0.042 0.167 0.125 0.083 0.000 24

SLB 0.000 0.000 0.000 0.000 0.083 0.333 0.083 0.000 0.000 0.000 0.000 0.042 0.042 0.000 0.125 0.000 0.125 0.083 0.042 0.042 24

GL 0.000 0.000 0.000 0.000 0.000 0.450 0.000 0.000 0.000 0.000 0.000 0.100 0.050 0.000 0.050 0.050 0.150 0.100 0.000 0.050 20

DK 0.000 0.000 0.000 0.000 0.028 0.278 0.000 0.056 0.056 0.000 0.028 0.056 0.139 0.000 0.083 0.111 0.028 0.056 0.056 0.028 36

FI 0.000 0.000 0.000 0.000 0.000 0.278 0.028 0.056 0.111 0.000 0.056 0.111 0.000 0.028 0.000 0.167 0.111 0.028 0.028 0.000 36

PT 0.000 0.000 0.000 0.000 0.067 0.300 0.000 0.067 0.067 0.033 0.000 0.033 0.000 0.033 0.033 0.200 0.067 0.000 0.033 0.067 30

WK 0.000 0.000 0.000 0.000 0.000 0.357 0.000 0.036 0.071 0.000 0.000 0.071 0.214 0.036 0.036 0.071 0.071 0.036 0.000 0.000 28

CHTENG 0.000 0.000 0.000 0.000 0.000 0.350 0.050 0.150 0.000 0.000 0.050 0.000 0.000 0.150 0.000 0.050 0.050 0.100 0.000 0.050 20
