# Supplementary material for: Evaluating the adaptive potential of the European eel: is the immunogenetic status recovering?
Source: PeerJ. 2016 Apr 11;4:e1868. doi: 10.7717/peerj.1868 (PMC4830236; doi:10.7717/peerj.1868)
Supplement: Data S3 [file peerj-04-1868-s003.rtf]

                      10        20        30        40        50        60                 
             ....|....|....|....|....|....|....|....|....|....|....|....|....|....
Anan-DXB0      QDLEFIDRYIFNKLEYARYNSTLNKFIGYTEHGVKNADRWNRDGEAERQHANLDSYCRHNAELSFN 
Anan-DXB1      ...............LL.............AL..Q....L....S..G..T...G...P......R 
Anan-DXB2      ......L..V......G........Y...........E.F......D...T...G...P......R 
Anan-DXB3      ....Y.S.........I..............L...H.EI.....S..QT.TY..G........... 
Anan-DXB4      ...............................F............G.........G........... 
Anan-DXB5      ..............................................D...T...G........... 
Anan-DXB6      ......S..V......L....................E.A.QE..P........G..KP......R 
Anan-DXB7      ...............................F.....EI..K..S..QT.....G........... 
Anan-DXB8      ......N.Q......FL..............L.......L..E..P........G..KP......R 
Anan-DXB9      ....Y.G..................V....D......E.L..............A...P...S... 
Anan-DXB10     ...............FL..............L.....EIF.Q..STD...I..EG........... 
Anan-DXB11     ......R.S....V..R........Y...........E.F....S.D...T...A...P......R 
Anan-DXB12     ....Y...F.......I....................E.F.Q..S..QA..D..R...Y....... 
Anan-DXB13     ...............................F.................................. 
Anan-DXB14     ................L........Y...........E.F....STD...N...AT..P....D.R 
Anan-DXB15     ......S.H......FLK......T......L.....KKL.QE..P....G...A...P......R 
Anan-DXB16     ....L.L..V.....LI........Y....AL.....E.F.Q..S..QA..G..V..K.......K 
Anan-DXB17     ...............................F............G..................... 
Anan-DXB18     ......L.S....V..R....................E.F......D...T...A...P......R 
Anan-DXB19     ...............FL..............L.......L..E..P....T..............R 
Anan-DXB20     ......L..V......L........Y...........E.F....Q..G......G..........R 
Anan-DXB21     ....Y.S.........I..............I...H.EI.....S..QT.TY..G........... 
Anan-DXB22     ......................................................G...LI...D.. 
Anan-DXB23     ......L..V...............Y...........E....E.A..G..T...A...P......R 
Anan-DXB24     ................L....................ETF....STD...N...AT..P....D.. 
Anan-DXB25     ......S..V......G....................E.F....S.....T.......P....... 
Anan-DXB26     ......L..V......G........Y...........E.F......DG..T...G...P......R 
Anan-DXB27     ................L........Y...........E.F......D...T...G........... 
Anan-DXB28     ...............LL........Y....AL..Q....L....S..S..T...G..KP....... 
Anan-DXB29     ......L.FV......G....................E.F......D.......G..........R 
Anan-DXB30     ................L....................A.F.......G..T...G...P..K.R.D 
Anan-DXB31     ....Y.L..V...............Y....D......E.F......D...T...A...P......R 
Anan-DXB32     ......R.S....V..R....................E.F....S.D...T...A...P......R 
Anan-DXB33     ................L........V...........E.F....S..S......A...P......G 
Anan-DXB34     ........H......FL..............L.......L..E..P....T...G..........R 
Anan-DXB35     ....Y.T.S....V..R........Y...........E.F......D....G......P....... 
Anan-DXB36     ...............................F......................G........... 
Anan-DXB37     ...............LL.............AL..Q....L....S.....T...GC.KP....... 
Anan-DXB38     ......S.........L........V...........E.F......D...I...G...P......R 
Anan-DXB39     ........H......FL..............L.......L..E..P....I...G..........R 
Anan-DXB40     ...............LL.............AL.....EK..Q..F..QT.....G..KP......R 
Anan-DXB41     ....Y.S..V......L....................E.F....Q..G...S..G...P......Q 
Anan-DXB42     ............................................RRD...T...G........... 
Anan-DXB43     ......H.S....V..R........Y...........E.F....S.D...T...A...P......R 
Anan-DXB44     ...............................F.....EI.....S..G..T...G...P......R 
Anan-DXB45     ...............................Y..............DS......G........... 
Anan-DXB46     ......L.S....V..R....................E.F....RRD...T...A...P......R 
Anan-DXB47     ...............................Y.............VD...T...G........... 
Anan-DXB48     ...............LI.............KF.....EI..K..F..QA.....G........... 
Anan-DXB49     ......S..................V....D......E.L......D...T...G...P......R 
Anan-DXB50     .................K................................................ 
Anan-DXB51     .........................V...........E.F....T...A.....A...P....A.. 
Anan-DXB52     ......................................................N........... 
Anan-DXB53     ......................................................G........... 
Anan-DXB54     ....Y.S..V......L........Y...........E.F....Q..GR..S......P......Q 
Anan-DXB55     H.....S..V......L........V...........E.F......D...V...G...P..K.R.D 
Anan-DXB56     ....Y.T.S....V..R........Y...........E.F......D....G..G...P....... 
Anan-DXB57     ...............LL........Y....AL..Q....L....S..S..T...V..KP....... 
Anan-DXB58     ......S.........L........A...........E.F......D...T...G...P......R 
Anan-DXB59     ...............LI.............KF.....EI..K..F..QT.....G..KP......R 
Anan-DXB60     .........................Y.....F...........E...S......G...PS...D.. 
Anan-DXB62     ......G.........L........V....D......E.L.Q....D...T...A...P...S... 
Anan-DXB63     ...............................F......................GS.......... 
Anan-DXB64     ......S..V......M........V...........E.F.......G......G........... 
Anan-DXB65     ................M........V.....I.....E.F....ST..E.....GT..P....... 
Anan-DXB66     ....Y.S........HI.............AL.....E...Q..F..QT.G.V.G...P....... 
Anan-DXB67     ........H......FL........Y.....F.......L..E.DP.H..T.V.V..KP......R 
Anan-DXB68     .........V...V..R......................L....S.D...T...G...P..K.C.D 
Anan-DXB69     ......S.S....V..R....................E.F....S.D...T...G...P......K 
Anan-DXB70     .........................V...........E.F....T...A.....A...P......R 
Anan-DXB71     ....L..........LL........Y....AL..Q....L....T..S......G..KP....... 
Anan-DXB72     ......L.FV......G....................E.F....RRD.......G..........R 
Anan-DXB73     ...............LI.............AL.....EI..K..STDQT.T..AG...P....... 
Anan-DXB74     .........................V...........E.F....T...A.....G...P......R 
Anan-DXB75     ....Y.L.FV......G....................E.F......D...T...A...P......R 
Anan-DXB76     ......L.S....V..R....................E.F......D.......A...P......R 
Anan-DXB77     H.....S..V......L........V...........E.F....S..QT.....G...P...S... 
Anan-DXB78     ................V..............F.....EI.....S..G..T...G...P......S 
Anan-DXB79     ....Y...........I....................ET..K..STDQT.T...G...P....... 
Anan-DXB80     ....Y...........I........Y...........E...Q..T..QT..D..G..KY....F.. 
Anan-DXB81     ...............FL..............L.....EIF.Q..STD...T..EG........... 
Anan-DXB82     ......N.H......FL........Y.....L.......L....GT............P......R 
Anan-DXB83     ....Y..........LI..............L.....EK..Q..T......D..G...S....A.. 
Anan-DXB84     ....Y...F.......I........Y...........E.F.Q..S..QA..D..R........... 
Anan-DXB85     ................M........V...........E.F....ST..E.....G..........S 
Anan-DXB86     ......S.........G....................E.F......D.......A...P..K.R.D 
Anan-DXB87     ...............LL.............AL.....E.A.QE..P....T.......P....R.D 
Anan-DXB88     ......L..V...............Y...........E...QE.A..G......G...P......R 
Anan-DXB89     ...............LL........Y....AL..Q....L....S..QT.T...G..KP......K 
Anan-DXB90     H.....S..V......L........I...........E.L....S..QT.....G...P...S... 
Anan-DXB91     ......L.S....V..R....................E.F..N...D...T...G...P..K.R.D 
Anan-DXB92     .........................Y.....F...............S......G........... 
Anan-DXB93     ...............VI.............AL.....EI..K..F..QT.G.V.G...P....... 
Anan-DXB94     ......S.H......FLK......T......L.....KKL.QE.RP....G...A...P......R 
Anan-DXB95     ...............LI..............L.....EI..K..ST.QA.....G...P..K.R.D 
Anan-DXB97     ......L..V...............Y...........E....E.A..G......G...P......R 
Anan-DXB98     ...............LI..............F.....EI..K..F..QT.....GV..P....D.. 
Anan-DXB99     H..............HI..............L.....EI..K..STDQT.T...A...P....... 
Anan-DXB100    .........................V...........E.F....T...E.....A...P......R 
Anan-DXB101    ......S.H......LM........Y....AI..Q....L....S..G......A...P..K.R.D 
Anan-DXB102    ....Y.I.........M........Y...........E.R.Q..S..QT.PY.............. 
Anan-DXB103    ......S........LL.............AL.....E.F....S..QT....EG..........D 
Anan-DXB104    ................L........V...........E.F....S..S......G...P......G 
Anan-DXB105    ................G....................E.F......D...T...G...P..K.R.D 
Anan-DXB106    ......L..V......L........Y...........A.F.......S......G...P..K.R.D 
Anan-DXB107    ...............................F.....EI........G..T...G...P......S 
Anan-DXB108    ......L..V.....LL.............AL..Q....L....S.....T...G...P..K.R.D 
Anan-DXB109    ......................................................GS.......... 
Anan-DXB110    ................I....................E...Q..STDQT.T...G..K........ 
Anan-DXB111    ................S.....................................G...P..K.R.D 
Anan-DXB112    ................M........V.....I.....E.F....ST..E.........P......R 
Anan-DXB113    ...............F...............L.......L....GT........G........... 
Anan-DXB114    ................I....................E...Q...........EG..........D 
Anan-DXB115    ................L..................D.K.A.QE..P........G...P....... 
Anan-DXB116    .........................V...........E.F....T...A.....A...P....T.R 
Anan-DXB117    .....................................E.F....ST..E.....G..........R 
Anan-DXB118    ....Y..........LI........Y.....L.....E...Q..SV..E..D..G........... 
Anan-DXB119    ...............LF........Y....AL..Q....L....S..S..T...G..KP....... 
Anan-DXB120    ...............LL.............AL.....E...Q..F..QT.....G..KP......R 
Anan-DXB121    ...............................F..............D...T...G........... 
Anan-DXB122    ................I..............F.......L..E..P........G..K.......R 
Anan-DXB123    ...............................F..............DSRN....G........... 
Anan-DXB124    ...............LI..............F.....EI..K..S.....T...GV..P.T....S 
Anan-DXB125    ...............................F..............DV.................. 
Anan-DXB126    ......S..V......L....................E.A.QE..P........G..KP.....LR 
Anan-DXB127    ................I..............F.......L..E..P........G..KP......R 
Anan-DXB128    ................I....................E...Q..STDQT.T...GV..P....I.R 
Anan-DXB129    ......S.H......FLK.............L.....K.M.QE...........G...P......R 
Anan-DXB130    ..V.L.I..S......I....................E...Q..F..QT.PY.............. 
Anan-DXB131    ......S..V......G....................E.F....S.....T.......P...S... 
Anan-DXB132    ......S.........L........A...........E.F....RRD...T...G...P......R 
Anan-DXB133    ....Y...........I....................ET..K..STDQT.T...G...P......D 
Anan-DXB134    H.....S..V......L........V...........E.F....RRD...V...G...P..K.R.D 
Anan-DXB135    ......L.S....V..R....................E.F....RRD.......A...P......R 
Anan-DXB136    ...............VM.............AL.....EIR.Q..SK.QER...EGT.KP....F.R 
Anan-DXB137    ......S.Q............................E...QE...........G...P....... 
Anan-DXB138    ....L..........VI.............AL.....EI..K..STDQA.T...G...P....... 
Anan-DXB139    ......S.........L........V...........E.F....RRD...I...G...P......R 
Anan-DXB140    ......R.S....V..R........Y...........E.F....ARD...T...A...P......R 
Anan-DXB141    ......N..V......I........................Q..SND...T...G...P......R 
Anan-DXB142    ...............LL.............AL..Q....L......D...T...G........... 
Anan-DXB143    ......L..V......I........Y..................S..G..T...A...P..K.R.D 
Anan-DXB144    ...............................F..............DV......G........... 
Anan-DXB145    ........H......FL..............L.......L..E.RQQQS.T...G..........R 
Anan-DXB146    ....Y.S.....N...V........Y.....L...D.E...K..S...A.....G...P......R 
Anan-DXB148    ...............LL.............AL.....E.A.QE..P....T...G...P....R.D 
Anan-DXB149    ...............................F..............D.......G........... 
Anan-DXB150    ......L..V...............Y....D........L....S..G..T...G...P....... 
Anan-DXB151    ......G.........L........V....D......E.L..E...D.......G...P....... 
Anan-DXB152    ....Y.S........HI.............AL.....EK..Q..F..QT.S.V.G...P....... 
Anan-DXB153    ......S........HI.............AL.....E...Q..T..QA..G..G..KP....T.. 
Anan-DXB154    ......S.H......FLK.............L.....KKM.QE...........G...P......R 
Anan-DXB155    ......S.........G....................E.F....RRD.......A...P..K.R.D 
Anan-DXB156    ......S..V...........................E...QE.AP....T...G...P......R 
Anan-DXB157    ......S..............................E...QE.AP....T...G...P......R 
Anan-DXB158    ....Y.S..V......L....................E.F....Q..GR..S......P......Q 
Anan-DXB159    ................I....................E...Q..FTDQT.T...G...P..K...D 
Anan-DXB160    ......S..M......L..................D.K.A.QE..P....G...G...P....... 
Anan-DXB161    ......H.Q......FL..............L.......L....GT.......NG..KP......R 
Anan-DXB162    ......S.........L.................N..K.A.QE..P....T...G...P..K.R.D 
Anan-DXB163    ......L..V.....LLI............AL..Q....L....S..G..T...G...P..K.R.D 
Anan-DXB164    ...............LI....................E...K..ST.QT.....GV..P..K.R.D 
Anan-DXB165    ...............LL........Y....AL..Q....L....S..S..T...G..KP......K 
Anan-DXB166    ......N........LI.............AL....VEI..K..STDQA.T...G...P....... 
Anan-DXB167    ....Y.S.....N...V........Y.....L...D.E...K..S...A.....G...P.V..Y.. 
Anan-DXB168    ......H.S....V..R........Y...........E.F....ARD...T...A...P......R 
Anan-DXB169    ......S.........I....................E...Q..STDQT.T...A...P..K.R.D 
Anan-DXB170    ......L.S....V..R....................E.F..N.RRD...T...G...P..K.R.D 
Anan-DXB171    ........H......FL........Y.....F.......L..E.ID.H..T.V.V..KP......R 
Anan-DXB172    ......S..............................E...QE..P....T...G...P....... 
Anan-DXB173    ..V.L.I..S......I....................E.R.Q..F..QT.PY.............. 
Anan-DXB174    ...............................F......................G...P....... 
Anan-DXB175    ........H......FL..............L.......L..E.RQQQS.I...G..........R 
Anan-DXB176    ....Y..........LM.............AL..Q....L....ST....T...GA..P......R 
Anan-DXB177    ......S..............................E...QE..P....T.......P....... 
Anan-DXB178    ......N.Q......FL..............L.......L..E..L..H.........P....F.R 
Anan-DXB179    ................G....................E.F......D.......A...P..K.R.D 
Anan-DXB180    H...............L........V.....N.....E.F....ST..E.....DS..P..K.R.D 
Anan-DXB181    ...............................Y............RID...T...G........... 
Anan-DXB183    ................I....................E...Q..FTDQT.T.V.G...P..K...D 
Anan-DXB184    ......L..V......G........Y...........E.F......D...T...A...P......R 
Anan-DXB185    ........H......FL........Y.....F.......L..E.IR.H..T.V.V..KP......R 
Anan-DXB186    ................S....................E.F......D...T...T...P......R 
Anan-DXB187    ......S..V......I....................K.F.QE.AP....T...G...P....... 
Anan-DXB188    ...............LI.............KF.....EI..K..F..QA.....G..........R 
Anan-DXB189    .........................Y.....F..............D.......G.......V... 
Anan-DXB191    ................L..............F.....E.A.QE..P....N...AT.KP....A.. 
Anan-DXB192    ...............................Y.............VD...T...G...X....... 
Anan-DXB193    ...............................F......................GF..P....... 
Anan-DXB194    ......G.........L........V....D......E.L..E.RRD.......G...P....... 
Anan-DXB195    ......S.Q................Y...........E...QE...........G...P....... 
Anan-DXB198    ......N.Q......FL..............L.......L..E.RP........G..KP......R 
Anan-DXB199    ..................C............................................... 
Anan-DXB200    ................I....................E...Q..FTDQT.T.......P..K...D 
Anan-DXB203    ...............................F..............DSRN................ 
Anan-DXB204    ................L........Y...........E.F......D.......AT..P....D.. 
Anan-DXB205    ................G....................E.F....RRD...T...G...P..K.R.D 
Anan-DXB208    ...............LI..............F.....EI..K..S..QT.....GV..P....D.. 
Anan-DXB209    ....Y.S.........I......S.......L...H.EI.....S..QT.TY..G........... 
Anan-DXB210    ......S.H......LM........Y....AI..Q....L....S..G......A...P..K.C.D 
Anan-DXB212    ................L....................A.F.....P....T...G...P..K.R.D 
Anan-DXB213    ......H.Q......FL..............L.......L.....R.QS....NG..KP......R 
Anan-DXB214    .........................Y.....F..............D.......G........... 
Anan-DXB215    .G....................................................G...LI...D.. 
Anan-DXB216    ......N.Q...R..FL..............L.......L..E..P........G..KP......R 
Anan-DXB217    ................L........Y...........E.F....PTD...N...AT..P....D.R 
Anan-DXB218    ........H......FL..............L.......L..E.RP....T...G..........R 
Anan-DXB219    ...............FL..............L.....EIF.Q..STD.R.I..EG........... 
Anan-DXB220    ....Y...........I........Y...........E...Q..T..QT..D..G..K........ 
Anan-DXB221    ........H......FL..............L.......L..E..P....T...G........G.R 
Anan-DXB222    ....Y.I.........I........Y...........E...Q..F..QT.PY............Y. 
Anan-DXB225    ........H......FL..............L.......L..E..P....I...G.....T....R 
Anan-DXB226    ......S..V......M........V...........E.F.......G......G....S...... 
Anan-DXB227    ...............LL.............AL.....E.A.QE.RQQQS.T...G...P....R.D 
Anan-DXB228    ......H.Q......FL..............L.......L..E.RP.QS.....G..KP......R 
Anan-DXB230    .........................Y.....F...............S......NT..P....T.. 
Anan-DXB231    .....T..........M........V.....I.....E.F....ST..E.....GT..P....... 
Anan-DXB232    ......H.Q......FLS.............L.......L..E.RQQQS.....G..KP......R 
Anan-DXB233    ................L........Y...........E.F....STD...S...AT..P....D.R 
Anan-DXB234    ......G.........L........V....D......EGL.Q....D...T...A...P...S... 
Anan-DXB235    ................I........S...........E...Q..STDQT.T...G..K........ 
Anan-DXB238    ................S....A...............E.F......D...T...T...P......R 
Anan-DXB239    ....Y.I.......D.I........Y...........E...Q..F..QT.PY.............. 
Anan-DXB241    ...............................F..............DV......GF..P....... 
Anan-DXB242    H.....S..V......L........V...........E.F..G.S..QT.....G...P...S... 
Anan-DXB243    ...............LL........Y....AL..Q....L....S..S..I...G..KP....... 
Anan-DXB244    ........H......FL..............L.......L..E.RQQES.T...G..........R 
Anan-DXB245    ................G....................E.F....RRD.......A...P..K.R.D 
Anan-DXB246    ......N........LI.............AL...D.EI..K..STDQA.T...G...P....... 

Amino Acid alignment of all Anan-DXBs MHC class II â identified in this study 
