# Supplementary material for: Evaluating the adaptive potential of the European eel: is the immunogenetic status recovering?
Source: PeerJ. 2016 Apr 11;4:e1868. doi: 10.7717/peerj.1868 (PMC4830236; doi:10.7717/peerj.1868)
Supplement: Table S1 — Pairwise distance matrix amongst sample locations. Above the diagonal are the values obtained with microsatellites, below the diagonal the ones obtained with mtDNA. Significant pairwise comparisons (p < 0.05) are depicted with *, or ** after correction for multiple comparison after (Narum, 2006). [file peerj-04-1868-s005.docx]

|  | **G**  **AD10** | **G**  **AD11** | **G**  **AD12** | **LC** | **BT** | **Q** | **G**  **WEG** | **G**  **VFRA** | **G**  **VSWE** | **G**  **TITA** | **G**  **BU** | **G**  **BNIL** | **G**  **OSPA** | **G**  **NIRL** | **G**  **EGER** | **BU** | **BL** | **SLC** | **LL** | **SLB** | **GL** | **DK** | **FI** | **Pt** | **Ger** | **G**  **TENG** |
| --- | --- | --- | --- | --- | --- | --- | --- | --- | --- | --- | --- | --- | --- | --- | --- | --- | --- | --- | --- | --- | --- | --- | --- | --- | --- | --- |
| **G_AD10** | - | 0.002 | 0.000 | 0.002 | 0.004 | 0.008 | 0.020 | 0.001 | 0.004 | 0.021 | 0.001 | 0.004 | -0.001 | 0.02* | 0.015 | 0.001 | 0.006 | 0.006 | 0.006 | 0.002 | 0.000 | 0.000 | 0.000 | 0.002 | 0.002 | 0.005 |
| **G_AD11** | 0.002 | - | 0.001 | 0.001 | 0.003 | 0.009 | 0.022* | 0.001 | 0.004 | 0.023 | -0.001 | 0.008 | 0.002 | 0.017 | 0.011 | 0.002 | 0.007 | 0.004 | 0.006 | -0.003 | -0.002 | 0.003 | -0.001 | -0.003 | -0.004 | 0.006 |
| **G_AD12** | -0.004 | -0.004 | - | 0.004 | 0.005 | 0.010* | 0.016 | 0.003 | 0.005 | 0.020 | -0.001 | 0.005 | 0.001 | 0.03** | 0.012 | 0.003 | 0.007 | 0.007 | 0.009* | -0.002 | 0.000 | 0.001 | 0.002 | -0.002 | -0.001 | 0.004 |
| **LC** | 0.018 | 0.007 | 0.010 | - | 0.004 | 0.010 | 0.022 | 0.015 | 0.005 | 0.034 | 0.004 | 0.012 | 0.018 | 0.023 | 0.010 | 0.003 | 0.010 | 0.009 | 0.009 | 0.003 | -0.005 | -0.001 | 0.006 | 0.007 | 0.000 | 0.011 |
| **BT** | -0.014 | -0.017 | -0.020 | -0.003 | - | 0.013 | 0.021 | 0.004 | 0.008 | 0.020 | 0.009 | 0.006 | -0.006 | 0.026** | 0.013 | 0.005 | 0.020** | 0.005 | 0.005 | 0.000 | -0.001 | 0.000 | 0.002 | -0.005 | 0.002 | 0.009 |
| **Q** | 0.016 | 0.049* | 0.029 | 0.103 | 0.029 | - | 0.023 | 0.000 | 0.014 | 0.030 | 0.001 | 0.009 | 0.012 | 0.036* | 0.027 | 0.007 | 0.017 | 0.016 | 0.007 | 0.014 | 0.013 | 0.007 | -0.001 | 0.014 | 0.008 | 0.016 |
| **G_WENG** | -0.038 | -0.035 | -0.045 | -0.004 | -0.071 | -0.006 | - | -0.001 | 0.030* | 0.051 | 0.007 | 0.012 | 0.015 | 0.067** | 0.020 | 0.020 | 0.030 | 0.013 | 0.023 | 0.019 | 0.017 | 0.013 | 0.026 | 0.014 | 0.015 | 0.013 |
| **G_VFRA** | 0.125 | 0.109 | 0.124 | 0.187 | 0.178* | 0.254 | 0.194 | - | 0.009 | 0.022 | 0.002 | 0.007 | 0.009 | 0.047 | 0.008 | -0.011 | 0.032 | -0.011 | 0.007 | 0.023 | 0.013 | -0.009 | 0.009 | 0.000 | 0.014 | -0.003 |
| **G_VSWE** | -0.003 | 0.012 | 0.001 | 0.076 | -0.009 | -0.021 | -0.044 | 0.167 | - | 0.018 | 0.006 | 0.016 | 0.017 | 0.019 | 0.010 | 0.005 | 0.017* | -0.003 | 0.012 | -0.010 | -0.005 | 0.005 | 0.002 | 0.005 | 0.001 | 0.000 |
| **G_TITA** | 0.062 | 0.009 | 0.034 | -0.034 | 0.031 | 0.244* | 0.035 | 0.089 | 0.142 | - | 0.021 | 0.018 | 0.002 | 0.066** | 0.028 | 0.017 | 0.048* | 0.031 | 0.030 | 0.026 | 0.022 | 0.031* | 0.028 | 0.030 | 0.026 | 0.026 |
| **G_BU** | 0.007 | -0.004 | -0.003 | -0.013 | -0.035 | 0.058 | -0.061 | 0.236 | 0.022 | 0.024 | - | 0.001 | 0.005 | 0.022 | 0.011 | 0.005 | 0.007 | 0.007 | 0.008 | -0.001 | 0.002 | 0.001 | -0.002 | -0.003 | -0.009 | 0.001 |
| **G_BNIRL** | -0.002 | -0.009 | -0.013 | 0.004 | -0.038 | 0.045 | -0.100 | 0.200* | 0.004 | 0.027 | -0.038 | - | 0.000 | 0.032* | 0.021 | 0.007 | 0.035** | 0.006 | 0.017 | 0.020* | 0.012 | 0.004 | 0.007 | 0.011 | 0.004 | 0.007 |
| **G_OSPA** | 0.010 | 0.020 | 0.018 | 0.070 | 0.031 | 0.090 | 0.035 | -0.136 | 0.048 | 0.048 | 0.121 | 0.071 | - | 0.026 | -0.001 | -0.007 | 0.028 | 0.018 | 0.009 | 0.004 | 0.013 | -0.009 | 0.004 | 0.004 | 0.010 | 0.014 |
| **G_NIRL** | -0.080 | -0.104 | -0.091 | -0.088 | -0.156 | 0.014 | -0.180 | 0.167 | -0.055 | -0.091 | -0.176 | -0.139 | 0.009 | - | 0.032 | 0.021 | 0.052** | 0.019 | 0.035* | 0.025 | 0.022 | 0.018 | 0.021 | 0.026* | 0.031* | 0.023 |
| **G_EGER** | -0.092 | -0.079 | -0.088 | -0.064 | -0.087 | -0.090 | -0.125 | -0.002 | -0.096 | -0.022 | -0.081 | -0.078 | -0.085 | -0.180 | - | 0.009 | 0.037* | 0.011 | 0.030 | 0.012 | 0.017 | 0.006 | 0.011 | 0.012 | 0.020 | 0.012 |
| **BU** | 0.035 | 0.057* | 0.040* | 0.075 | 0.014 | 0.003 | -0.022 | 0.401 | 0.027 | 0.241* | 0.016 | 0.018 | 0.211 | -0.030 | -0.022 | - | 0.011 | 0.001 | 0.005 | -0.006 | -0.002 | 0.001 | 0.004 | 0.002 | 0.004 | 0.006 |
| **BL** | -0.022 | -0.027 | -0.025 | -0.042 | -0.035 | 0.061 | -0.034 | 0.052 | 0.016 | -0.050 | -0.003 | -0.011 | -0.081 | -0.100 | -0.107 | 0.081 | - | 0.010 | 0.012 | 0.006 | 0.010 | 0.005 | 0.013 | 0.010 | 0.014 | 0.011 |
| **SLC** | 0.021 | 0.033 | 0.024 | 0.122 | 0.004 | 0.022 | -0.032 | 0.241 | -0.038 | 0.183* | 0.058 | 0.019 | 0.081 | -0.033 | -0.032 | 0.055 | 0.043 | - | 0.014 | 0.000 | -0.004 | 0.007 | 0.006 | -0.003 | 0.005 | 0.005 |
| **LL** | 0.024 | -0.004 | 0.010 | -0.034 | 0.010 | 0.135 | 0.008 | 0.065 | 0.080 | -0.101 | 0.002 | 0.012 | 0.024 | -0.083 | -0.070 | 0.140* | -0.048 | 0.127 | - | 0.000 | 0.008 | 0.007 | 0.008 | 0.009 | 0.006 | 0.010 |
| **SLB** | -0.011 | -0.013 | -0.011 | -0.005 | -0.014 | 0.061 | -0.020 | 0.046 | 0.011 | -0.010 | 0.023 | 0.000 | -0.052 | -0.071 | -0.089 | 0.010* | -0.069 | 0.020 | -0.017 | - | -0.003 | -0.008 | 0.005 | 0.001 | 0.004 | 0.000 |
| **GL** | 0.003 | -0.013 | -0.009 | 0.007 | -0.028 | 0.078* | -0.055 | 0.113 | 0.020 | -0.020 | -0.012 | -0.028 | 0.000 | -0.120 | -0.072 | 0.069 | -0.034 | 0.035 | -0.017 | -0.01 | - | 0.003 | 0.005 | -0.002 | 0.004 | 0.004 |
| **DK** | 0.013 | 0.005 | 0.005 | 0.064* | -0.007 | 0.050 | -0.043 | 0.124 | -0.013 | 0.047 | 0.012 | -0.008 | 0.047 | -0.084 | -0.067 | 0.083* | 0.010 | -0.013 | 0.037 | 0.007 | -0.016 | - | -0.002 | -0.006 | -0.003 | 0.006 |
| **FI** | 0.004 | -0.013 | -0.007 | 0.003 | -0.015 | 0.070* | -0.031 | 0.076 | 0.014 | -0.025 | -0.016 | -0.011 | 0.020 | -0.101 | -0.099 | 0.088* | -0.034 | 0.047 | -0.032 | -0.011 | -0.024 | -0.011 | - | 0.003 | -0.003 | 0.003 |
| **Pt** | 0.003 | -0.011 | -0.007 | -0.001 | -0.031 | 0.076* | -0.046 | 0.188 | 0.009 | 0.008 | -0.020 | -0.022 | 0.073 | -0.114 | -0.061 | 0.053 | -0.032 | 0.013 | -0.003 | -0.019 | -0.019 | -0.003 | -0.014 | - | 0.003 | 0.003 |
| **Ger** | 0.019 | -0.001 | 0.007 | -0.044 | -0.008 | 0.121* | -0.009 | 0.157 | 0.069 | -0.069 | -0.014 | -0.002 | 0.062 | -0.101 | -0.056 | 0.096 | -0.048 | 0.103 | -0.046 | -0.017 | -0.008 | 0.042 | -0.014 | -0.021 | - | 0.004 |
| **G_TENG** | 0.010 | 0.015 | 0.010 | 0.090 | 0.013 | 0.013 | -0.030 | 0.051 | -0.024 | 0.098 | 0.040 | 0.015 | -0.004 | -0.050 | -0.105 | 0.098 | 0.020 | 0.009 | 0.056 | 0.017 | 0.006 | -0.019 | -0.002 | 0.041 | 0.077 | - |
