# Supplementary material for: Evaluating the adaptive potential of the European eel: is the immunogenetic status recovering?
Source: PeerJ. 2016 Apr 11;4:e1868. doi: 10.7717/peerj.1868 (PMC4830236; doi:10.7717/peerj.1868)
Supplement: Table S2 — Estimates of effective population size (Ne) for each of the replicates, with respective confidence intervals. [file peerj-04-1868-s006.docx]

| **Replicates** | **Ne (Pcrit 0.05)** | **CI (0.05)** |
| --- | --- | --- |
| SL 1 | 424.3 | *(408.8-440.1)* |
| SL 2 | -1564.4 | *-* |
| SL 3 | -485.3 | *-* |
| SL 4 | 447.3 | *(432.0-462.9)* |
| SL 5 | -1410.5 | *-* |
| SL 6 | 470.4 | *(454.1-486.9)* |
| SL 7 | -3366.6 | *-* |
| SL 8 | 625.2 | *(601.7-649.1)* |
| SL 9 | -1116.5 | *-* |
| SL 10 | -3155 | *-* |
| GL 1 | 1426.1 | *(1377.5 -1475.5)* |
| GL 2 | -2284.7 | *-* |
| GL 3 | 1032.9 | *(996.9-1069.5)* |
| GL 4 | 1673.3 | *(1616.2 -1731.4)* |
| GL 5 | 11871 | *(11461.2-12287.8)* |
| GL 6 | -674.6 | *-* |
| GL 7 | -3340.6 | *-* |
| GL 8 | 1209.7 | *(1166.9 -1253.3)* |
| GL 9 | 655.9 | *(632.7-679.5)* |
| GL 10 | 2708.9 | *(2608.2-2811.6)* |
