# Supplementary material for: Evaluating the adaptive potential of the European eel: is the immunogenetic status recovering?
Source: PeerJ. 2016 Apr 11;4:e1868. doi: 10.7717/peerj.1868 (PMC4830236; doi:10.7717/peerj.1868)
Supplement: Table S3 — - nHap, number of haplotypes; S, segregation sites; Hd, Haplotype diversity; π, nucleotide diversity; k, average number of differences; nr alleles/ind, average number alleles per individual with respective standard error (se); distnt, average nucleotide distance per individual with respective standard error (se). [file peerj-04-1868-s007.docx]

| **Population** | **nAlleles** | **nIndividuals** | **nHap** | **S** | ***h*** | ***π*** | **nr alleles/ind** | **se** | **dist_nt** | **se** |
| --- | --- | --- | --- | --- | --- | --- | --- | --- | --- | --- |
| Adour2010 | 70 | 24 | 45 | 88 | 0.979 | 0.1173 | 2.917 | 0.282 | 0.104 | 0.013 |
| Adour2011 | 81 | 24 | 47 | 95 | 0.982 | 0.12555 | 3.375 | 0.300 | 0.141 | 0.010 |
| Adour2012 | 69 | 20 | 57 | 94 | 0.985 | 0.12146 | 3.450 | 0.380 | 0.108 | 0.012 |
| Burrishole_juv | 56 | 14 | 38 | 84 | 0.975 | 0.11058 | 4.000 | 0.392 | 0.108 | 0.012 |
| BannLower | 29 | 11 | 24 | 80 | 0.99 | 0.13198 | 2.636 | 0.472 | 0.078 | 0.019 |
| BannToome | 38 | 16 | 32 | 96 | 0.98 | 0.13255 | 2.375 | 0.287 | 0.102 | 0.019 |
| Burrishole | 45 | 17 | 33 | 80 | 0.979 | 0.12417 | 2.647 | 0.308 | 0.105 | 0.016 |
| Denmark | 46 | 17 | 31 | 83 | 0.969 | 0.12058 | 2.706 | 0.361 | 0.099 | 0.015 |
| Finland | 62 | 18 | 43 | 83 | 0.985 | 0.12635 | 3.444 | 0.506 | 0.141 | 0.011 |
| GlynnLagoon | 55 | 19 | 37 | 84 | 0.97 | 0.12972 | 2.895 | 0.374 | 0.089 | 0.015 |
| LC | 46 | 16 | 33 | 88 | 0.979 | 0.12539 | 2.875 | 0.352 | 0.117 | 0.015 |
| LL | 33 | 12 | 24 | 82 | 0.949 | 0.11316 | 2.750 | 0.305 | 0.115 | 0.019 |
| Portugal | 46 | 13 | 33 | 90 | 0.979 | 0.11261 | 3.538 | 0.584 | 0.120 | 0.014 |
| Quoile | 22 | 11 | 15 | 77 | 0.938 | 0.11787 | 2.000 | 0.270 | 0.096 | 0.021 |
| SLB | 51 | 14 | 38 | 91 | 0.978 | 0.1176 | 3.643 | 0.401 | 0.127 | 0.015 |
| SLC | 28 | 11 | 22 | 84 | 0.972 | 0.12844 | 2.545 | 0.366 | 0.100 | 0.021 |
| Viskan_juv | 56 | 15 | 40 | 93 | 0.985 | 0.13439 | 3.733 | 0.565 | 0.125 | 0.020 |
| Germany | 153 | 54 | 87 | 99 | 0.988 | 0.12299 | 2.833 | 0.218 | 0.082 | 0.008 |
