# Supplementary material for: Evaluating the adaptive potential of the European eel: is the immunogenetic status recovering?
Source: PeerJ. 2016 Apr 11;4:e1868. doi: 10.7717/peerj.1868 (PMC4830236; doi:10.7717/peerj.1868)
Supplement: Table S4 — ω = (dN∕dS), the ratio of non-synonymous (dN) per synonymous (dS) substitutions; LRT, likelihood ratio statistic for β + = α (null or no selection) vs β + unrestricted (alternative or episodic selection). [file peerj-04-1868-s008.docx]

| **CODEML** | **estimate ω >1** | **proportion sites ω>1** | **codon sites ω>1** |
| --- | --- | --- | --- |
| M1vsM2 | 3.899 | 0.167 | 7;17;26;40;45;46;51;55 |
|  |  |  | 59;64;66 |
| M8vsM7 | 3.191 | 0.167 | 7;17;26;40;45;46;51;55 |
|  |  |  | 59;64;66 |
| **Datamonkey** | **estimate LTR** | **proportion sites p(LTR)<0,001** | **codon sites p(LTR)<0,001** |
| MEME | 48.139 | 0.409 | 7;9;10;16;17;18;19;22;26 |
|  |  |  | 35;38;39;40;42;44;45;46; |
|  |  |  | 48;49;53;54;56;58;59;63;64;65 |
