# Supplementary figures and images for: Evaluating the adaptive potential of the European eel: is the immunogenetic status recovering?
Source: PeerJ. 2016 Apr 11;4:e1868. doi: 10.7717/peerj.1868 (PMC4830236; doi:10.7717/peerj.1868)

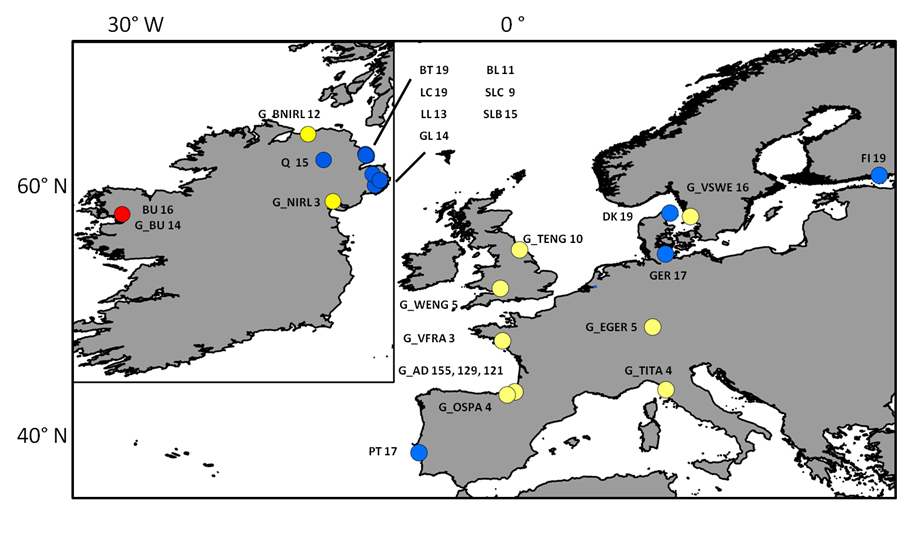

Supplement: Figure S1 — Sampling sites and respective number of individuals (n) collected for each site and used for mtDNA and microsatellite analyses. Blue dots represent locations where “silver eels” where collected, yellow dots represent locations where “glass eels” where collected and the red dot represents location where both were collected. The G_p prefix stands for “glass eels”. Three numbers for the location of G_AD (Adour) depicts the n for three sampling events: G_AD2010, G_AD2011 and G_AD2012 respectively. The remaining acronyms have the following meaning: LC (Larne Lagoon), BT (Bann Toome), Q (Quoile), BU (Burrishole), BL (BannLower), SLC (LoughComber), DK (Denmark), LL (LarneLagoon), SLB (Boretree), GL (Glynn Lagoon), FI (Finland), PT (Portugal), Ger (Germany), G_BU (Burrishole), G_BNIRL (Northern Ireland), G_VSWE (Sweden), G_TENG (England), G_EGER (Germany), G_OSPA (Spain), G_VFRA (France), G_WENG (England), G_TITA (Italy), G_NIRL (Northern Ireland). [file peerj-04-1868-s009.png]

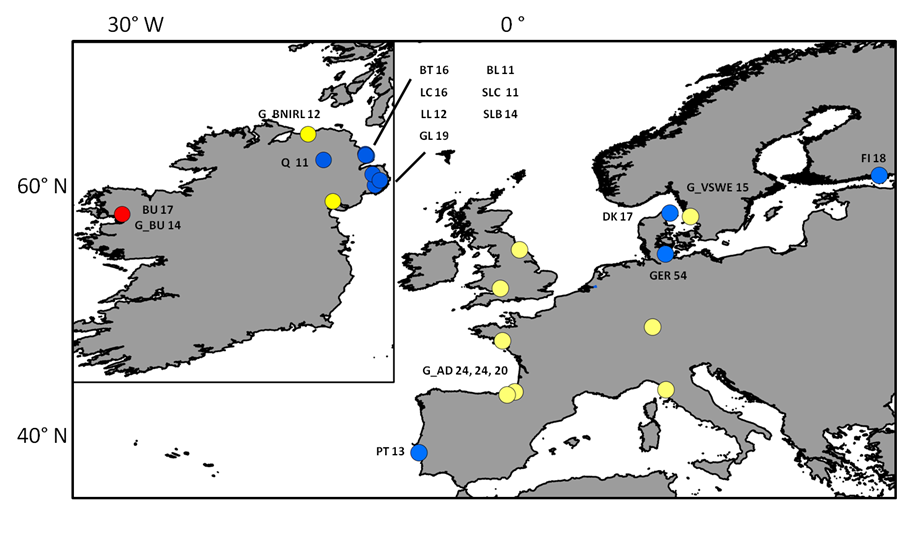

Supplement: Figure S2 — Labels are the same as in the previous picture. [file peerj-04-1868-s010.png]

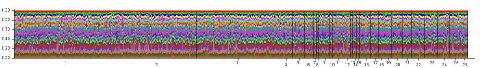

Supplement: Figure S3 [file peerj-04-1868-s011.png]

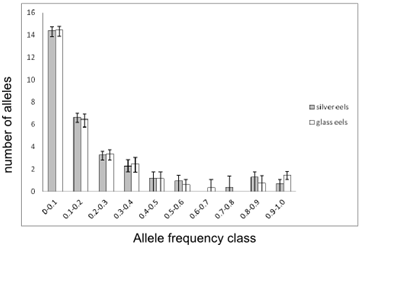

Supplement: Figure S4 — Above is shown the average distributions of allele’s frequency classes for “silver eels” (grey bars) and “glass eels” (open bars). Error bars represents the maximum and minimum number of alleles observed amongst replicates. Values on the Y-axis were obtained by multiplying the number of alleles, (k), with the frequency of the respective class. For purposes of visualization, all values were transformed to their square roots. [file peerj-04-1868-s012.png]

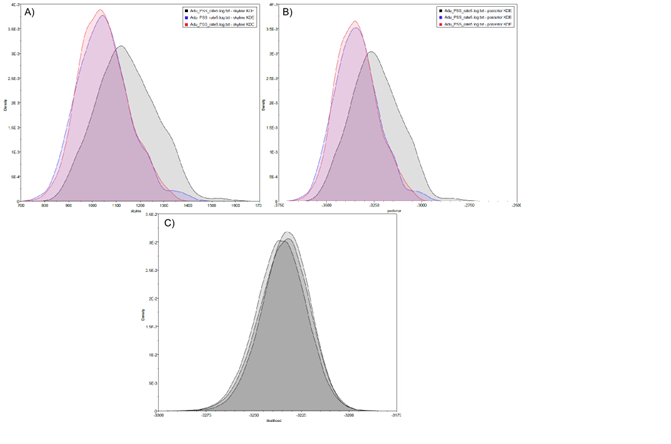

Supplement: Figure S5 — Both the skyline (A), posterior (B) and likelihood (C) overlap, conferring statistical support for the shape of the Bayesian plots produced with MHC data (Figs. 4 and 5 of the main text). [file peerj-04-1868-s013.png]
